# African Swine Fever Virus Undergoes Outer Envelope Disruption, Capsid Disassembly and Inner Envelope Fusion before Core Release from Multivesicular Endosomes

**DOI:** 10.1371/journal.ppat.1005595

**Published:** 2016-04-25

**Authors:** Bruno Hernáez, Milagros Guerra, María L. Salas, Germán Andrés

**Affiliations:** Centro de Biología Molecular Severo Ochoa, Consejo Superior de Investigaciones Científicas and Universidad Autónoma de Madrid, Madrid, Spain; University of Muenster - ZMBE, GERMANY

## Abstract

African swine fever virus (ASFV) is a nucleocytoplasmic large DNA virus (NCLDV) that causes a highly lethal disease in domestic pigs. As other NCLDVs, the extracellular form of ASFV possesses a multilayered structure consisting of a genome-containing nucleoid successively wrapped by a thick protein core shell, an inner lipid membrane, an icosahedral protein capsid and an outer lipid envelope. This structural complexity suggests an intricate mechanism of internalization in order to deliver the virus genome into the cytoplasm. By using flow cytometry in combination with pharmacological entry inhibitors, as well as fluorescence and electron microscopy approaches, we have dissected the entry and uncoating pathway used by ASFV to infect the macrophage, its natural host cell. We found that purified extracellular ASFV is internalized by both constitutive macropinocytosis and clathrin-mediated endocytosis. Once inside the cell, ASFV particles move from early endosomes or macropinosomes to late, multivesicular endosomes where they become uncoated. Virus uncoating requires acidic pH and involves the disruption of the outer membrane as well as of the protein capsid. As a consequence, the inner viral membrane becomes exposed and fuses with the limiting endosomal membrane to release the viral core into the cytosol. Interestingly, virus fusion is dependent on virus protein pE248R, a transmembrane polypeptide of the inner envelope that shares sequence similarity with some members of the poxviral entry/fusion complex. Collective evidence supports an entry model for ASFV that might also explain the uncoating of other multienveloped icosahedral NCLDVs.

## Author Summary

Virus entry is a crucial initial event for productive infection, being therefore a potential target for antiviral strategies. African swine fever virus (ASFV) is the causative agent of a frequently fatal swine disease for which there is no vaccine. ASFV belongs to the superfamily of nucleocytoplasmic large DNA viruses (NCLDV), which are among the most complex viruses known. ASFV genome locates at a core structure that is wrapped by two lipid membranes separated by an icosahedral protein capsid. Here we have dissected the internalization process of ASFV into host macrophages. Our results indicate that ASFV uses two alternative endocytic mechanisms, clathrin-mediated endocytosis and macropinocytosis, an ongoing process in macrophages. Once internalized, ASFV particles move to multivesicular endosomes, where they undergo a disassembly process leading to the loss of the two outermost layers. This exposes the inner viral envelope, which fuses to the limiting endosome membrane to deliver the viral core into the cytosol. ASFV penetration depends on acidic pH and on the inner envelope viral protein pE248R. Our findings point to an internalization model that could also explain the uncoating of other icosahedral enveloped NCLDVs. Also, they provide new cellular and viral targets for the development of antiviral strategies against ASFV.

## Introduction

Most viruses take advantage of existing cellular endocytic pathways to enter their host cells [1–4]. Once internalized, virus particles move through a dynamic network of endocytic vesicles, which undergo gradual sorting and complex maturation events. Endosome maturation, in turn, triggers conformational changes and dissociation events in the incoming viruses, which ultimately lead to the delivery of the viral genome and associated proteins into the cytoplasm. In general, while endocytosed non-enveloped viruses are able to penetrate the limiting endosomal membrane by lysis or pore formation [5], enveloped viruses fuse with it to be released into the cytoplasm [6].

The repertoire of endocytic mechanisms used by viruses includes clathrin-mediated endocytosis (CME), caveolar/raft-dependent endocytosis, macropinocytosis, phagocytosis and less-characterized non-clathrin, non-caveolae pathways [3]. CME is the best characterized and common of the endocytic pathways employed by small and intermediate viruses [7]. CME involves the receptor-dependent internalization of virus particles through the formation of a clathrin coat underneath the plasma membrane [7]. Clathrin-coated pits bud into the cytoplasm after a scission event assisted by the GTPase dynamin. The resulting coated vesicles, with an internal diameter of 60–200 nm, deliver the viral cargo into peripheral early endosomes, which eventually mature into perinuclear late endosomes and then into lysosomes. Importantly, endosome maturation provides to the incoming viruses with specific cues, such as pH acidification or proteolytic processing of viral proteins, required for viral uncoating and fusion. Accordingly, virus penetration can occur at different endosome types, including early and late endosomes, and even lysosomes [8].

Macropinocytosis involves a non-selective uptake of extracellular fluid and particles driven by actin-dependent evaginations of the plasma membrane [9, 10]. It leads to the formation of large, uncoated endocytic vesicles known as macropinosomes, which typically range from 0.2 to 10 μm. Macropinosomes undergo a maturation program reminiscent of that of classical endosomes, with which they ultimately intersect [11]. Macropinocytosis is constitutively active in macrophages and dendritic cells, but it is also triggered by some growth factors, as well as by an increasing number of viruses [12–15].

In this report we have investigated the entry pathway of African swine fever virus (ASFV), a genetically and structurally complex, multienveloped DNA virus. ASFV causes a highly lethal hemorrhagic fever of domestic pigs, for which there is no vaccine or antiviral strategy available. Currently, the disease is endemic in sub-Saharan Africa and recent outbreaks have been reported in many countries of Eastern Europe and the Caucasus [16]. ASFV is the sole member of the Asfarviridae family of nucleocytoplasmic large DNA viruses (NCLDV). The genome is a double-stranded DNA that contains more than 150 genes [17, 18]. The extracellular virus particle, with an overall icosahedral shape and an average diameter of 200 nm, is organized in several concentric layers [19, 20]. It consists of an inner viral core, which contains a central genome-containing nucleoid coated by a thick protein layer referred to as core shell. The viral core is successively enwrapped by an inner lipid envelope, a protein capsid and an outer lipid membrane.

ASFV mainly infects cells of the swine immune system such as monocytes and macrophages. DNA replication and virus morphogenesis occurs within discrete cytoplasmic areas close to the nucleus referred to as viral factories [21, 22]. In these assembly sites, intracellular particles acquire their inner envelope from the endoplasmic reticulum [23, 24]. The outer membrane is taken by budding at the plasma membrane during virus exit [25]. Both intracellular and extracellular ASFV forms are infectious [26].

Early studies showed that extracellular ASFV enters host cells by receptor-mediated endocytosis [27, 28] and that the virus internalization is pH-dependent [27, 29]. The mechanism of ASFV uptake remains, however, controversial, as recent reports have provided highly contradictory evidence. Whereas some studies stated that ASFV uses clathrin-dependent endocytosis [30, 31] as the primary entry pathway, another work established that ASFV triggers its own uptake by macropinocytosis [32]. Strikingly, each study excluded the alternative mechanism. Once internalized, incoming viruses locate to the endolysosomal pathway from where they presumably exit into the cytoplasm after uncoating and fusion [33, 34]. However, the precise manner in which extracellular ASFV particles become uncoated and fuse through one of their two membranes still remains unresolved.

In the present report, we have dissected the internalization pathway of extracellular ASFV in swine macrophages by using fluorescence and electron microscopy approaches, as well as flow cytometry-based assays in combination with different entry inhibitors. We have found that ASFV enters via both constitutive macropinocytosis and clathrin-mediated endocytosis. Then, the incoming virus moves to late endosomes, where it undergoes a pH-driven loss of the two outermost layers. Finally, the fusion of the inner viral envelope with the limiting endosomal membrane delivers naked cores into the cytosol.

## Results

### ASFV enters swine macrophages by clathrin-mediated endocytosis and macropinocytosis

The endocytic mechanism responsible for ASFV entry is controversial. Whereas some works [30, 31] stated that ASFV enters through clathrin- and dynamin-mediated endocytosis, another report concluded that ASFV induces its own internalization by macropinocytosis [32]. Importantly, none of these works used purified virions in their assays but clarified infections supernatants, which contain huge amounts of cell debris when examined at the EM level ([35], see also S1 Fig). Moreover, this cell debris was found to attach to the cells, which may significantly interfere with the uptake assays and/or their interpretation. In this report, we have revisited the mechanism of ASFV entry by using extracellular particles purified by Percoll-density gradient sedimentation ([35], S1 Fig). Also, at variance with previous reports, we have used a number of direct assays to estimate the uptake of ASFV into swine macrophages, its primary target cell.

**Fig 1:**
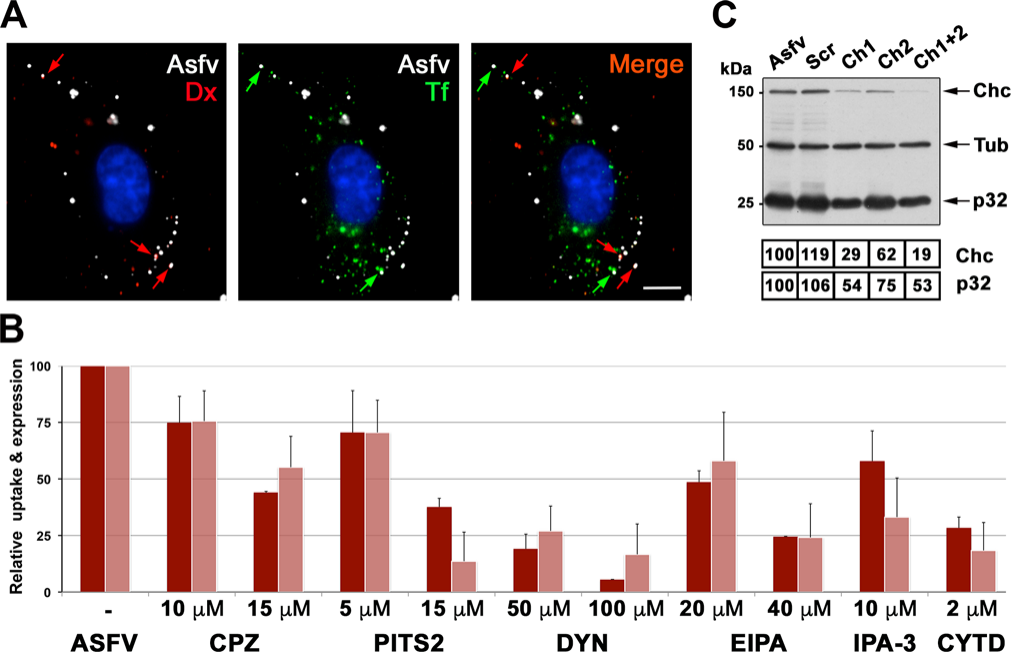
ASFV uses both clathrin-mediated endocytosis and macropinocytosis to enter swine macrophages. **A**) ASFV-infected swine macrophages (MOI 10) were pulsed for 10 min at 37°C transferrin conjugated to AF488 and 10-kDa dextran conjugated to AF555. After fixation, ASFV particles were labeled with an antibody against capsid protein p72 followed by an Alexa 647-conjugated secondary antibody. Note that incoming ASFV particles colocalized with either dextran, used as a macropinocytosis tracer (red arrows), or CME marker transferrin (green arrows). Bar, 5 μm. **B**) Macrophages pre-treated for 15 min with a number of inhibitors for clathrin-dependent endocytosis (chlorpromazine (CPZ), pitstop 2 (PTS2) and dynasore (DYN)) and macropinocytosis (EIPA, IPA-3 and cytochalasin D (CYT.D)) were incubated with DiD-labeled fluorescent ASFV particles (MOI 5) for 30 min. Then, the cells were incubated for an additional 30 min period in the presence of inhibitors and analyzed for virus uptake by flow cytometry (dark red colums). In a second set of experiments, macrophages were treated as above but infection was extended to 2.5 hpi to allow detection of the expression of early viral protein p32 by immunoblotting (light red columns). Data are expressed as relative fluorescence or p32 expression compared to a control infection (mean of three independent experiment ± SD). **C**) The expression of clathrin heavy chain (Chc) was silenced in macrophages by using two different siRNAs (Ch1, Ch2). Then, macrophages were infected with ASFV (MOI 5) for 2.5 h and analyzed by immunoblotting for Chc, ASFV protein p32 and ß-tubulin (Tub). No siRNA-treated infected cells (Asfv) and a scrambled (Scr) siRNA were used as controls. Numbers under the blot image indicate normalized expression levels for Chc and p32.

As a first approach, we analyzed the possible colocalization of incoming ASFV particles with fluorescent markers for CME and macropinocytosis. To this aim, ASFV-infected swine macrophages were pulsed for 10 min at 37°C with Alexa fluor (AF) 488-labeled transferrin, a CME marker, and 10-kDa dextran-AF555, a fluidic phase marker. As shown in Fig 1A, the incoming virus particles (immunolabeled for capsid protein p72) were found to colocalize with vesicles containing either dextran (~68%) or transferrin (~25%), or both (~7%), thus suggesting that the ASFV can use both endocytic mechanisms, being macropinocytosis the main entry route.

Next we investigated the impact on ASFV entry of diverse pharmacological drugs known to prevent either CME or macropinocytosis. To this purpose, macrophages were infected for 30 min at 37°C with ASFV particles, previously labeled with the fluorescent lipophilic dye DiD, in the presence of both classes of inhibitors. Then, non-adsorbed particles were washed out and incubation with inhibitors was prolonged for an additional 30 min period. Virus entry was quantified by flow cytometry using non-treated ASFV-infected cells as a control. As shown in Fig 1B, ASFV uptake was significantly affected in a dose-dependent manner by macropinocytosis inhibitors like EIPA, which blocks vacuolar Na ^+^ /H ^+^ antiporters, IPA-3, an inhibitor of p21-activated kinase 1 (Pak1) and cytochalasin D, an inhibitor of actin polymerization. Importantly, the clathrin inhibitors chlorpromazine (CPZ) and pitstop2 (PTS2) as well as the dynamin inhibitor dynasore (DYN) also strongly reduced the ASFV uptake. To analyze the effect of these inhibitors on viral expression, the incubation of infected cells with the drugs was prolonged for 2.5 h to allow the detection of early virus protein p32. As shown in Fig 1B, ASFV infection was also strongly reduced by both classes of endocytosis inhibitors. Since some CME inhibitors have been reported to have serious side effects on the actin cytoskeleton and to affect fluidic-phase uptake [36] [37], we compare the effects of CPZ, PTS2, DYN and hyperosmotic sucrose on the infection of ASFV and vaccinia virus (VACV), a virus entering by macropinocytosis [14], in swine macrophages. As shown in S2 Fig, all the inhibitors significantly reduced ASFV infection whereas VACV infection was not inhibited at all by CPZ and hyperosmotic sucrose. However, the inhibitors DYN and PTS2 moderately affected VACV infection (by ~ 10% and 20%, respectively), which suggests they could also exert a limited side effect on the macropinocytic entry of ASFV.

Finally, we analyzed the effect of a combination of CME (CPZ) and macropinocytosis (EIPA) inhibitors on ASFV uptake and viral expression in macrophages. As shown in S3 Fig, the results indicate a nearly complete inhibition of virus entry and viral expression, thus supporting the notion that both CME and macropinocytosis explain most if not all the observed ASFV uptake.

In a different approach, we analyzed the effect of silencing the expression of clathrin heavy chain (Chc) on ASFV uptake and infection. To this purpose, macrophages were transfected with two siRNAs targeting Chc and then infected with ASFV for 2.5 h. As shown in Fig 1C, the partial silencing of Chc (by 38–81%) was accompanied by a significant reduction of viral p32 expression (by 25–47%). Collectively, these results indicate that ASFV particles can use both clathrin-dependent endocytosis and macropinocytosis to enter and productively infect host macrophages.

**Fig 2:**
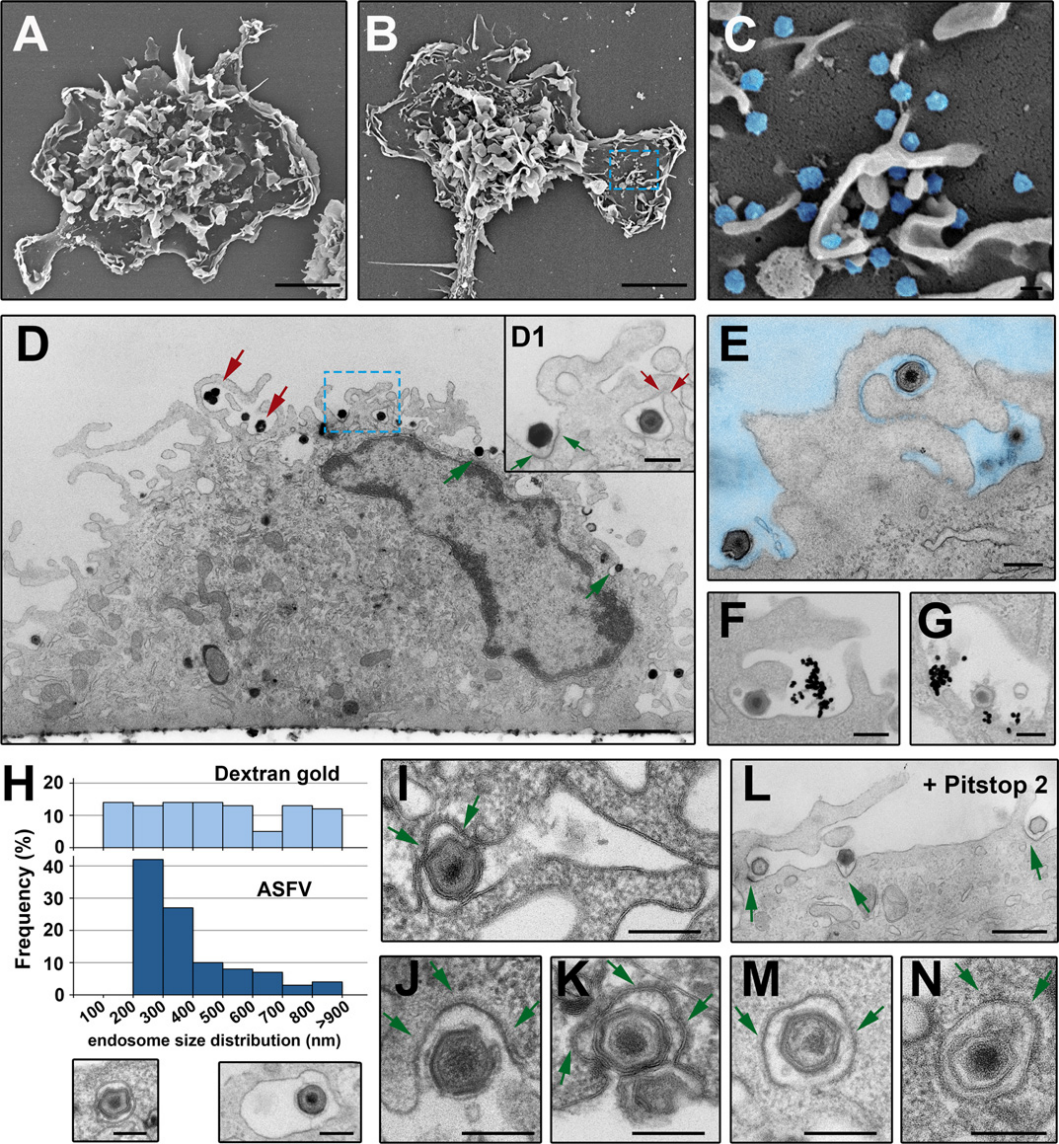
ASFV endocytosis at the ultrastructural level. Mock (**A**) and ASFV-infected (**B** and inset **C**) (MOI 100) swine macrophages were fixed at 20 mpi and analyzed by field emission scanning EM. The characteristic cell membrane protrusions of monocyte/macrophage lineage can be visualized in both conditions. ASFV particles (depicted in blue in inset **C**) are detected on flat membrane areas and also in the interstices of the membrane evaginations. **D**-**E**, **I**-**J**, **L-M**) ASFV-infected macrophages (MOI 100) were examined at 10 mpi by transmission EM after fixation. Panel **D** shows a side view of an infected macrophage. Note that ASFV particles seem to be endocytosed by macropinocytic-like membrane protrusions (red arrows) as well as by membrane invaginations characteristic of clathrin-dependent endocytosis (green arrows). Inset **D1** shows a detail of panel D at higher magnification. **E** shows a virion apparently engulfed by macropinocytosis. **F-G**) Macrophages were incubated with both ASFV and dextran-coated 30-nm gold particles for 7 min at 37°C. **F** shows macropinocytic-like uptake of both ASFV and dextran-gold particles and **G** shows colocalization at a putative macropinosome. **H**) Size distribution histogram of endosomes containing either dextran-coated 30-nm gold particles (upper) or ASFV particles (lower) after 7 min of incubation. **I-J, L-M**) ASFV uptake by clathrin-coated pits (**I, J** and **K**) and coated vesicles (**M** and **N**). The clathrin coats are indicated by arrows. **L**) ASFV-infected macrophages were incubated for 15 min with 15 μM Pitstop 2. Arrows indicate virions at coated pits. Bars, 200 nm except for panels A and B (5 μm), D (1 μm) and L (500 nm).

Next, we investigated the ASFV uptake at the ultrastructural level by using different electron microscopy (EM) strategies. First, we compared non-infected with ASFV-infected macrophages at 20 mpi by field emission scanning EM. As shown in Fig 2A, mock-infected cells displayed typical features of the monocyte/macrophage lineage. They consist of abundant and irregularly shaped, large cell membrane protrusions at central cell areas, and relatively flat regions outlined by membrane ruffles at the periphery. This complex cell topography was not altered after ASFV infection (Fig 2B). Interestingly, adsorbed ASFV particles could be easily detected on smooth membrane areas as well as at the interstices between membrane evaginations (Fig 2C). In a different approach, ASFV-infected macrophages were analyzed by using transmission EM. To facilitate the visualization of virus uptake and to minimize morphological alterations related to conventional EM processing, the infected macrophages were *in situ* fixed, dehydrated and flat-embedded before ultrathin sectioning in a plane orthogonal to the substrate (Fig 2D and 2M). Inspection of infected macrophages at 10 mpi evidenced clear cues of the two different entry mechanisms (Fig 2D). On the one hand, many virus particles seemed to be engulfed by a macropinocytic-like process involving large membrane protrusions, which eventually collapsed back to the plasma membrane (red arrows in Fig 2D and inset D1, see also Fig 2E). On the other hand, virus particles were also frequently detected in dense membrane invaginations, reminiscent of clathrin-dependent endocytosis (green arrows in Fig 2D and inset D1).

To further explore the uptake of ASFV, swine macrophages were infected for 7 min in the presence of dextran-coated gold nanoparticles of 30 nm, which were used as a fluid-phase marker. Fig 2F and 2G illustrates the joint uptake of incoming virions and dextran-gold particles into large uncoated electronlucent vesicles reminiscent of macropinosomes. In general, macropinosomes, which are among the largest pinocytic vesicles, range from 200 nm to several microns [9]. In the swine macrophage model, the size distribution of dextran-containing vesicles spanned from 100 to 1000 nm, with an average diameter of 490 ± 260 nm (n>100). It should be noted that the actual macropinosome dimensions are probably underestimated in this assay due to the frequent detection of gold particles within clathrin-coated vesicles (~ 10% of the positive vesicles). Interestingly, the size distribution of virus-containing endosomes (mean:380 ± 150 nm, n>100) showed a narrower profile (Fig 2H). Indeed, more than 65% of particles were detected in vesicles within a 200–400 nm range, which strongly suggests that macropinocytosis alone cannot explain the uptake of ASFV.

The relevance of CME in ASFV uptake was evidenced by the frequent presence of virions inside clathrin-coated pits (Fig 2I and 2J) and derived coated vesicles (Fig 2L and 2M). Indeed, more than 15% of the cell-adsorbed virions (n>200) at 10 mpi were found inside coated pits. Interestingly, while the mean size of coated vesicles in non-infected macrophages was 105 ± 30 nm, those containing virus particles were 245 ± 20 nm. This indicates that clathrin-coated pits can be deformed to fit large particles, as has been described for vesicular stomatitis virus [38]. CME was even more evident after incubation of infected macrophages with Pitstop 2 (Fig 2K), a clathrin inhibitor that “freezes” coated pit structures. Finally, additional evidence for CME was obtained by immunogold labeling of clathrin heavy chain on cryosections of ASFV-infected Vero cells (S4 Fig). Altogether, different EM approaches demonstrated at the ultrastructural level that ASFV uses both macropinocytosis and clathrin-dependent endocytosis to enter the macrophage.

### Purified ASFV does not induce macropinocytosis

**Fig 3:**
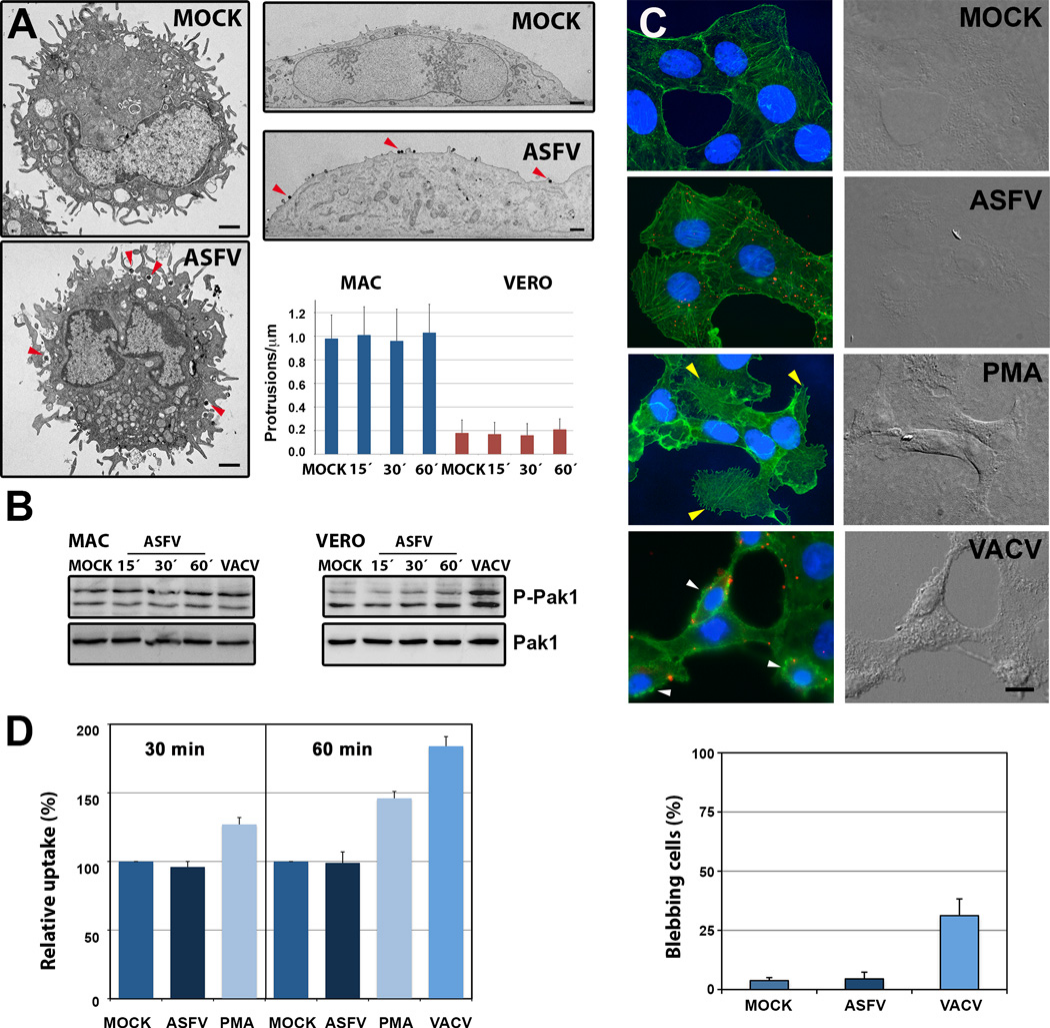
ASFV does not induce macropinocytosis. **A**) Mock and ASFV-infected (MOI 100) macrophages were fixed and processed by transmission EM at 15, 30 and 60 mpi. Representative micrographs of Mock and ASFV-infected macrophages (left panels) and Vero cells (upper right panels) at 15 mpi are shown. ASFV particles are indicated by red arrowheads. Bars, 1 μm. Cells treated as before were quantified for the membrane protrusion density (number of protrusions > 200 nm per μm of plasma membrane from 50 cell profiles). Data display mean values and SD from a representative experiment. **B**) Macrophages (MAC) or Vero cells were infected (MOI 5; E70 or BA71V strains, respectively) for the indicated times and the phosphorylation levels of Pak1 (Thr423) were determined by immunoblotting. As a control of Pak activation, VACV-infected cells (MOI 5) were analyzed at 30 mpi. Levels of total Pak1 are shown below. **C**) Mock- and DiD-labeled ASFV-infected Vero cells (MOI 10) were incubated at 37°C for 15 min, fixed, stained for actin (green) and cell nucleus (Hoechst 33258, blue), and analyzed by wide-field fluorescence and DIC microscopy. As control, cells treated with 200 nM PMA, or infected with VACV (MOI 10) are shown. Bar, 5 μm. Incoming virus particles are shown in red. The yellow arrowheads indicate spike-like protrusions in PMA-treated cells while the white arroheads indicate blebbing in VACV-infected cells. Bar, 5 μm. In the bottom panel, the percentage of cells displaying blebbing in control cells (MOCK) or after infection with ASFV or VACV for 15 min is shown. Data indicate mean ± SD from two independent experiments. **D**) Mock- and ASFV-infected Vero cells (MOI 10) were pulsed for 30 and 60 min at 37°C with 10-kDa dextran-AF-488. As a control, cells treated with 200 nM PMA (30 and 60 min) or infected with VACV (60 min) were analyzed in parallel. Dextran uptake was quantified by flow cytometry and normalized to unstimulated cells (mean percentage ± SD of three independent experiments).

Since macropinocytosis is a constitutively activated process in macrophages [9], it is not surprising that ASFV uses this non-selective endocytic mechanism. However, it has been reported that ASFV is able to induce its own macropinocytic uptake [32], as described for other viruses [39]. We therefore revisited this issue by examining the ability of purified extracellular ASFV particles to induce the formation of typical macropinocytic structures such as lamellipodia, circular ruffles, or blebs in the target cells. Scanning (Fig 2A–2C) and transmission EM (Fig 3A, left panels) of swine macrophages infected with ASFV at 15–30 mpi did not reveal any significant induction of membrane protrusions at the cell surface, which were already abundantly present in non-infected cells. The induction of actin-driven membrane evaginations is dependent on the activation by phosphorylation of Pak1 kinase [39]. We therefore addressed this possibility by analyzing the levels of phosphorylated Pak1 in infected macrophages. As shown in Fig 3B (left panel), neither ASFV or VACV, a virus inducing its own entry by macropinocytosis [14] significantly triggered Pak1 phosphorylation in macrophages along the analyzed interval (0–60 mpi).

Since macropinocytosis is highly activated in macrophages, a possible induction by ASFV could be obscured by the overall constitutive activity. To overcome this possible concern, we analyzed the capacity of ASFV to trigger macropinocytosis in Vero cells, a kidney epithelial-like monkey lineage displaying wide smooth cell surfaces and occasional microvilli. As shown in Fig 3A, transmission EM of Vero cells infected with ASFV at 15–60 mpi did not reveal any significant induction of membrane protrusions at the cell surface. Consistent with this, field emission scanning EM (S5 Fig) of Vero cells infected for 20 mpi with massive amounts of ASFV particles (about 10,000 virus particles/cell) did not reveal significant induction of membrane protrusions either. In another approach, we analyzed the ability of ASFV to induce membrane ruffling, blebbing or actin remodeling. To this purpose, Vero cells were infected with DiD-labeled ASFV at 37°C for 15 min. As a positive control, cells were either treated with phorbol esther PMA or infected with VACV. Cells were fixed and analyzed by DIC and wide-field fluorescence microscopy. No significant induction of membrane ruffling, blebbing or actin reorganization was observed in ASFV-infected cells (Fig 3C). At variance, PMA induced extensive actin reorganization, which was paralleled by membrane ruffling and spike-like protrusions, while VACV-infected cells displayed significant blebbing. Consistent with these results, ASFV infection did not trigger significant Pak1 activation (Fig 3B, right panel) in Vero cells whereas VACV significantly increased the levels of phosphorylated Pak1.

If ASFV triggered macropinocytosis for its internalization, an increase in fluid-phase uptake would be expected [39]. To test this possibility, the uptake of fluorescent 10-kDa dextran was analyzed in serum-starved Vero cells after ASFV infection. Quantification by flow cytometry (Fig 3D) after a 30- or 60-min pulse showed no significant increase whereas PMA treatment or VACV infection increased dextran uptake by more than 45 and 85%, respectively, at 60 min. Altogether, these results support the notion that purified extracellular ASFV particles do not significantly induce macropinocytosis.

### ASFV moves across the entire endolysosomal system

**Fig 4:**
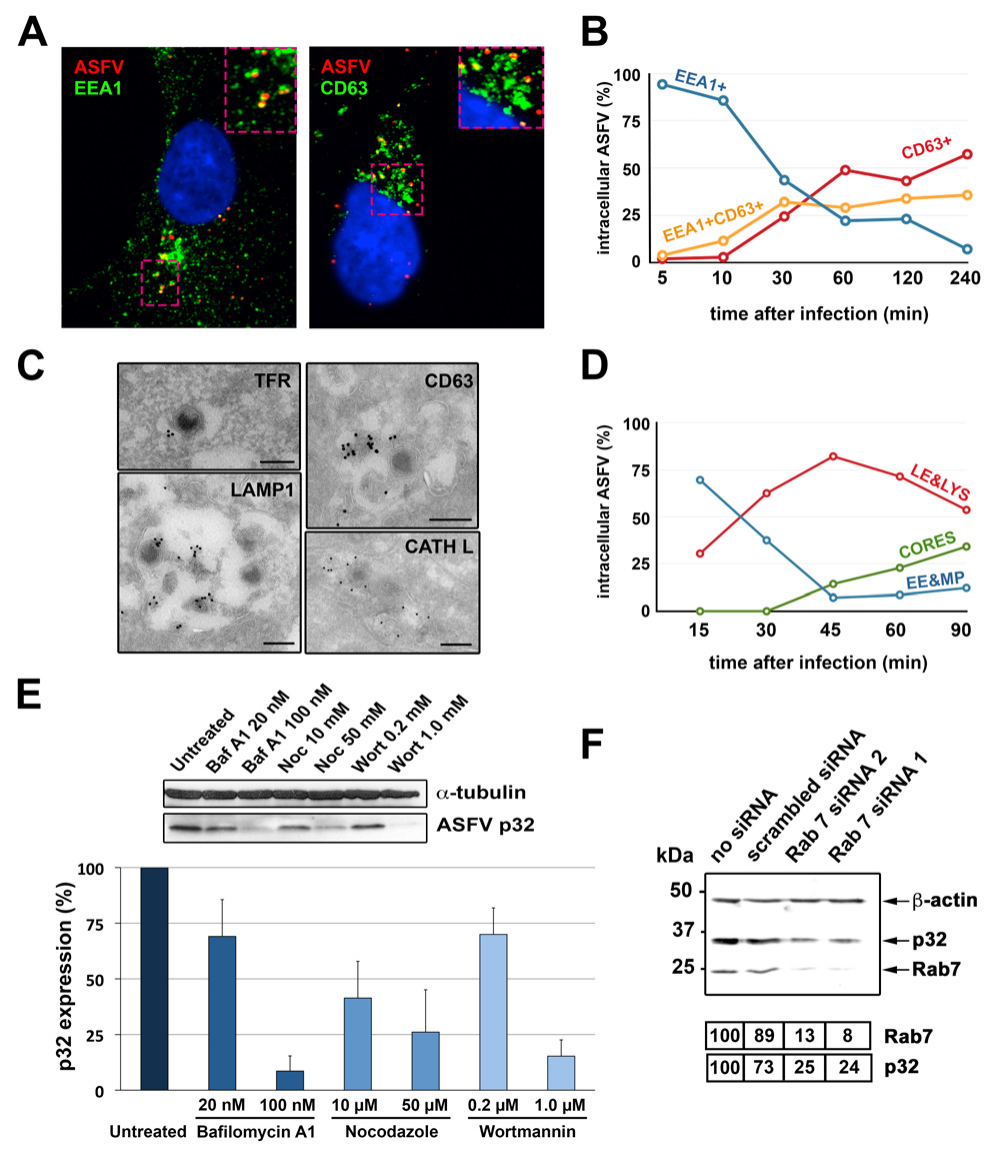
Transport of incoming ASFV along the endocytic pathway. **A**-**B**) ASFV-infected Vero cells (MOI 10) were analyzed at the indicated times by immunofluorescence detection of viral protein p17, early endosome/macropinosome marker EEA1 and late endosome/lysosome marker CD63. **A**) Colocalization of incoming viruses (red) with EEA1+ vesicles (green) at 10 mpi (left) or with CD63+ vesicles (green) at 30 mpi (right). **B**) Quantification of ASFV particles colocalizing with either EEA1, CD63 or both markers at the indicated times. **C**) Immunoelectron microscopy of incoming ASFV particles within endocytic vesicles labeled for early endosomal marker TFR, late endosomal/lysosomal markers CD63 and LAMP1, and lysosomal cathepsin L (CATH L). Immunogold labeling was performed on thawed cryosections of ASFV-infected cells using the indicated antibodies and protein A-gold (15 nm) conjugates. Bars, 200 nm. **D**) Transport of incoming ASFV in swine macrophages. Infected macrophages (MOI 100) were processed by EM at the indicated times. For each time, intracellular particles were classified and quantified according to their presence within early endosomes and macropinosomes (EE&MP), late endosomes and lysosomes (LE&LYS) or at the cytosol (CORES). **E**) ASFV-infected macrophages (E70, MOI 5) were treated for 2.5 h with inhibitors of endosome maturation (Baf A1, nocodazole and wortmannin) and analyzed for expression of viral protein p32 by immunoblotting (upper panel). Graphic shows percentage of p32 expression (mean ± SD from triplicate samples) referred to control, non-treated cells. **F**) COS-1 cells were transfected with two different siRNAs (1 and 2) targeted to Rab7, a scrambled siRNA or not treated (no siRNA). Then, cells were infected with ASFV (MOI 5) for 5 h. Expression of proteins p32, Rab7 and ß-actin was analyzed by immunoblotting. Numbers under the blot image indicated normalized expression levels for Rab7 and p32.

We next investigated the transport of incoming ASFV particles within endocytic vesicles. As a first approach, the localization of ASFV inside early and late endocytic vesicles was investigated by immunofluorescence microscopy in Vero cells at different times of infection. As shown in Fig 4A and 4B, virus particles immunolabeled for inner envelope protein p17 were predominantly detected at early times (from 5 to 30 mpi) at EEA1+ vesicles, consistent with their presence within early endosomes or macropinosomes. At later times (from 30 to 240 mpi), incoming particles were increasingly colocalized with perinuclear CD63+ vesicles, indicative of late endosomes and lysosomes. The trafficking of ASFV particles across the entire endocytic network was further confirmed by immunogold labeling on cryosections of ASFV-infected Vero cells. As shown in Fig 4C, virus particles were detected within vesicles labeled for the early endocytic marker transferrin receptor (TFR) as well as inside multivesicular and multilamellar endosomes labeled with late endosomal/lysosomal markers CD63, Lamp1 and cathepsin L.

In a different approach, the endocytic transport of ASFV was monitored *in vivo* by using transfected COS-1 cells transiently expressing gfp-conjugated versions of early (Rab5) and late (Rab7) endocytic markers. Subsequent infection with fluorescent DiD-labeled particles allowed the colocalization of ASFV particles with Rab5+ vesicles at early times (15 mpi, S1 and S2 Videos) and with Rab7+ vesicles at later times (30 mpi, S3 Video). Furthermore, correlative light-electron microscopy confirmed the presence of virus particles inside both early, Rab5+ and late, Rab7+ endocytic structures (S6 Fig, S4 Video). Interestingly, while virus particles detected inside Rab5+ early compartments looked essentially intact, those found into Rab7+ multivesicular endosomes and lysosomes seemed to be partially disrupted, suggesting that they underwent an uncoating process (S6 Fig).

**Fig 5:**
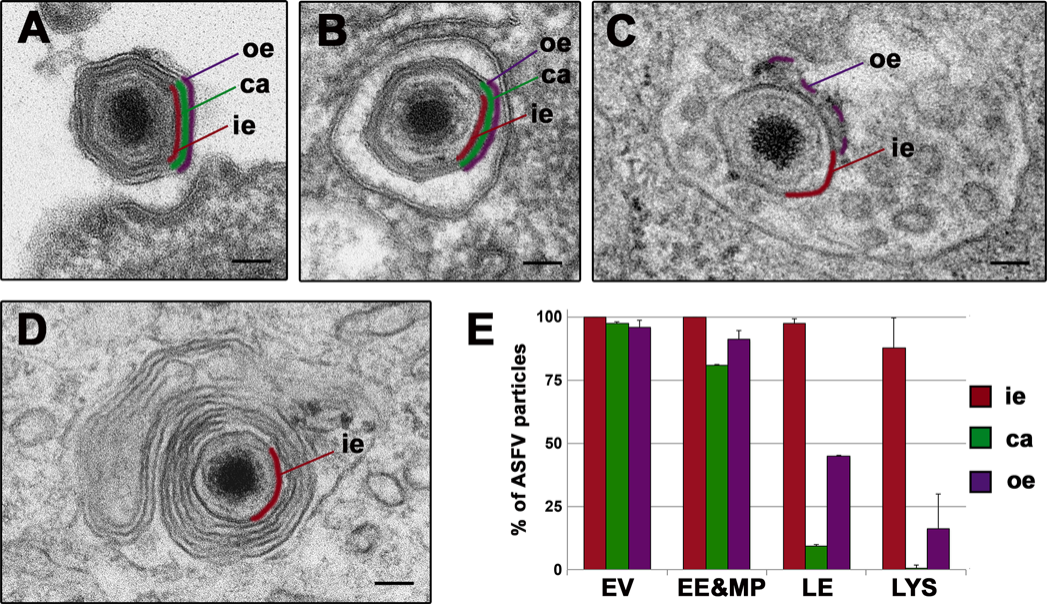
ASFV disassembly in swine macrophages. Macrophages were infected with ASFV (MOI 100) for 10, 15, 30, 45, 60 and 90 min and processed by EM. **A-D**) Selected EM images of the endosomal transport and the disassembly process undergone by ASFV. After endocytosis, incoming extracellular particles (**A**) are first detected (10–15 mpi) inside early endosomes (**B**) or macropinosomes keeping their structure nearly intact. Later (30–45 mpi), particles are predominantly found at multivesicular late endosomes (**C**), where a significant proportion loses their protein capsid (depicted in green) and the outer envelope (purple). Finally (45 mpi onwards), a fraction of particles reach lysosome-like structures (**D**) where most of them appear as a dense cores enwrapped by the inner envelope (red). Bars, 50 nm. **E**) Quantification of the ASFV disassembly. Incoming virus particles were classified according to their endocytic compartment (EE&MP: early endosomes and macropinosomes; LE: late multivesicular endosomes, LYS: lysosomes) and then, according to their layer content (ie: inner envelope, ca: capsid, oe: outer envelope). Data are expressed as percentages (mean ± deviation of duplicate experiments) of particles with a given domain inside each compartment. As a reference, extracellular virions (EV) attached to the cell surface were also quantified. More than 100 particles per compartment and experiment were analyzed. Note that the loss of the capsid and the outer envelope occurs essentially at multivesicular late endosomes and lysosome-like vesicles.

Next we analyzed the time course of ASFV transport in their natural host, the swine macrophage. Due to the lack of suitable endocytic swine markers, this study was performed by standard EM using morphological criteria as described in Materials and Methods. Incoming ASFV particles were first detected (10 mpi) inside electronlucent vesicles ranging from 200 to 500 nm, which contained none or a few intraluminal vesicles (Fig 5B). These structures were interpreted as primary endocytic vesicles as well as early endosomes/macropinosomes. At later times (30 mpi onwards), virus particles were mostly detected in larger endosomes ranging from 300 to 800 nm, which contained abundant intraluminal vesicles (Fig 5C) and/or membrane sheets (Fig 5D). These vesicles were interpreted as late endosomes and lysosomes. A third category of subviral particles consisting of naked cores (characterized in more detail later) was detected from 45 mpi onwards in the cytosol. Fig 4D shows the time distribution of the incoming ASFV particles in the three above defined categories. Altogether, these data demonstrate the transit of ASFV from early to late endocytic structures in swine macrophages.

### ASFV infection depends on maturation to late endosomes

The endosomal trafficking is paralleled with a maturation program that entails profound changes in the localization, morphology and biochemical composition of the endocytic vesicles [40]. Such changes, in turn, provide the endocytosed virions with the necessary cues for the activation of an uncoating program that culminates in the penetration of the genome-containing subviral particle through the endosomal membrane [3].

To explore the relationship between endosome maturation and ASFV penetration in macrophages, we first analyzed the impact of different inhibitors known to interfere with endosomal maturation. To this aim, ASFV-infected macrophages were incubated with bafilomycin A1 (Baf A1), an inhibitor of vacuolar H+/ATPase pump that prevents endosome acidification and transport from early to late endosomes, nocodazole, a microtubule-depolymerizing drug that disturbs early endosome movement, and wortmannin, a PI 3-kinase inhibitor affecting macropinocytic entry and also the early endosome fusion and consequent maturation. As shown in Fig 4E, ASFV infection, as judged by the expression of the early viral protein p32, was significantly impaired in a dose-dependent manner by all tested inhibitors. Moreover, when the expression of Rab7, a key regulator in the maturation of early to late endosomes, was transiently silenced in COS-1 cells by means of specific siRNAs, ASFV infection was strongly impaired (Fig 4F). Altogether, these results strongly indicate that productive ASFV infection in swine macrophages depends on endosomal transport and maturation to late endocytic vesicles.

### ASFV disassembly involves the loss of the outer envelope and the protein capsid within multivesicular endosomes

To dissect the uncoating process of ASFV, we performed quantitative EM analysis of the incoming virus particles detected inside the different endocytic vesicles from 15 to 90 mpi. To this aim, internalized virus particles were first classified according to the intracellular compartment and then quantified according to their layer composition. Fig 5E shows the frequency distribution of virus particles containing the inner envelope, the capsid or the outer envelope for each endocytic compartment. As reference, extracellular virions (EV) attached to the cell surface were also quantified. As shown in Fig 5B, most virus particles inside early endocytic vesicles looked nearly intact and similar to adsorbed extracellular virions (Fig 5A and 5E). In contrast, most virus particles (>85%) inside multivesicular endosomes (Fig 5C and 5E) lack the protein capsid while a significant proportion (>50%) had lost the outer membrane. Interestingly, when the outer membrane was present, it often appeared broken and partially dissociated from the underlying particle (Fig 5C). Finally, virus disassembly was virtually completed at lysosomal-like structures containing multilamellar membranes (Fig 5D and 5E). Similar results were obtained in ASFV-infected Vero cells (S7 Fig). Collectively, our data indicate that ASFV disassembly, which involves disruption of the outer membrane and icosahedral capsid, occurs at late, multivesicular endosomes.

### ASFV fuses with multivesicular endosomes through the inner viral membrane

**Fig 6:**
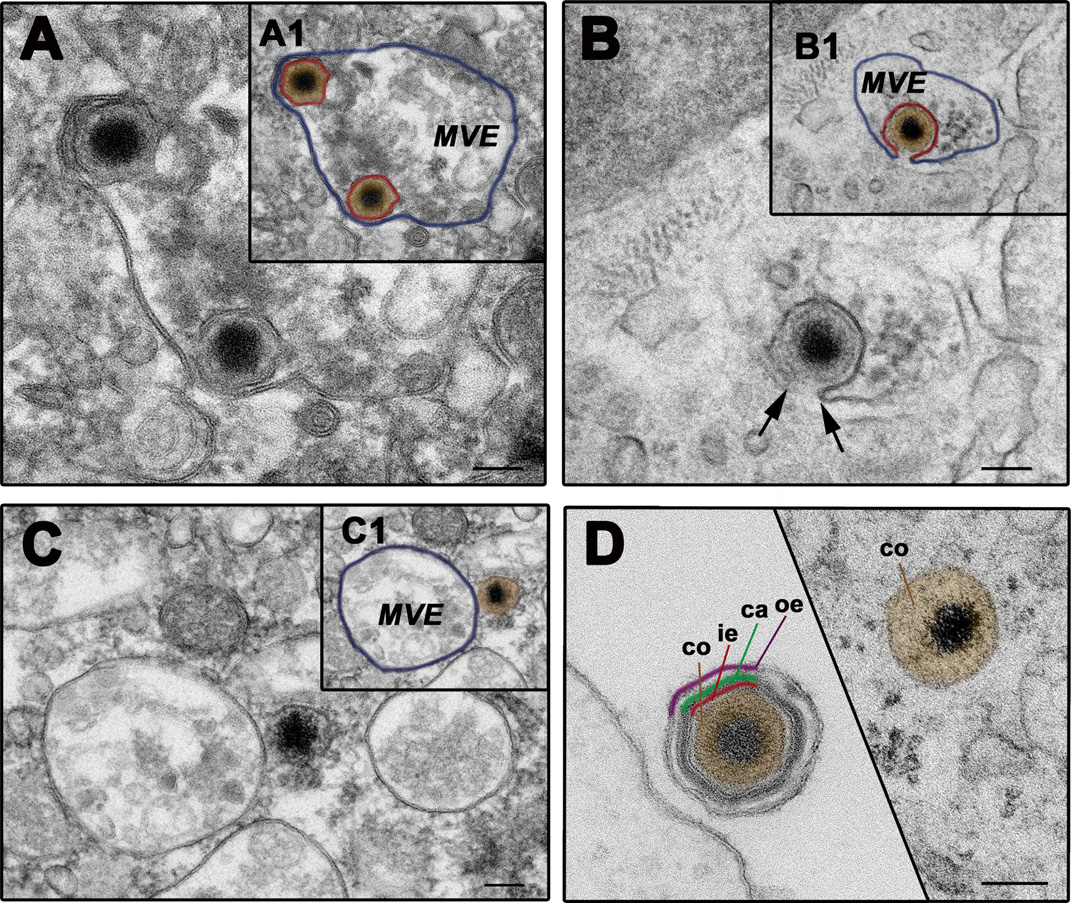
ASFV fuses through the inner envelope at late multivesicular endosomes. ASFV-infected swine macrophages (MOI 100) were analyzed by EM at 90 mpi. **A-D**) Representative EM images of the fusion process. After the loss of protein capsid and outer viral membrane, the inner viral envelope becomes exposed, interacting with the luminal face of the limiting membrane of multivesicular late endosomes (MVE) (**A**). Then, the inner envelope fuses with the endosomal membrane (**B**) and delivers naked cores into the cytosol (**C**). As a result of the disassembly and fusion events, extracellular particles lose the three domains surrounding the virus core before reaching the cytosol **(D**). Insets A1, B1 and C1 show lower magnification images of panel A, B and C, respectively. To facilitate the interpretation, the inner viral envelope is depicted in red, the virus core in brown and the endosomal membrane in purple. Bars, 100 nm.

After the removal of the two outermost layers, the inner viral envelope becomes exposed. Therefore, a fusion event involving that membrane would permit the viral genome to be delivered into the cytosol. To examine this possibility, we inspected virus-containing multivesicular endosomes at 90 mpi by EM (Fig 6). Interestingly, a significant proportion of virus particles were tethered through their inner envelope to the luminal face of the limiting endosomal membrane (Fig 6A). Importantly, we also could visualize virus particles that seemed to undergo a fusion process between the exposed inner envelope and the endosomal membrane (Fig 6B). Finally, cytosolic subviral particles, identified as naked cores, were frequently detected in close proximity or, even in tight contact, to late endosomes and lysosomes (Fig 6C). The cytosolic cores appeared enlarged when compared to those of intact extracellular particles (150 nm ± 12 vs. 120 ± 8 nm; Fig 6D). The overall ultrastructure was, however, similar, consisting of a dense, genome-containing nucleoid wrapped by a thick core shell, which in turn was outlined by a thin dense layer (S7J Fig). Altogether, these observations indicate that ASFV uses the inner envelope to fuse with the limiting membrane of late multivesicular endosomes and, maybe, of later endocytic vesicles. As a result, genome-containing naked cores are delivered into the cytosol.

### ASFV disassembly and uncoating depend on acidic pH

**Fig 7:**
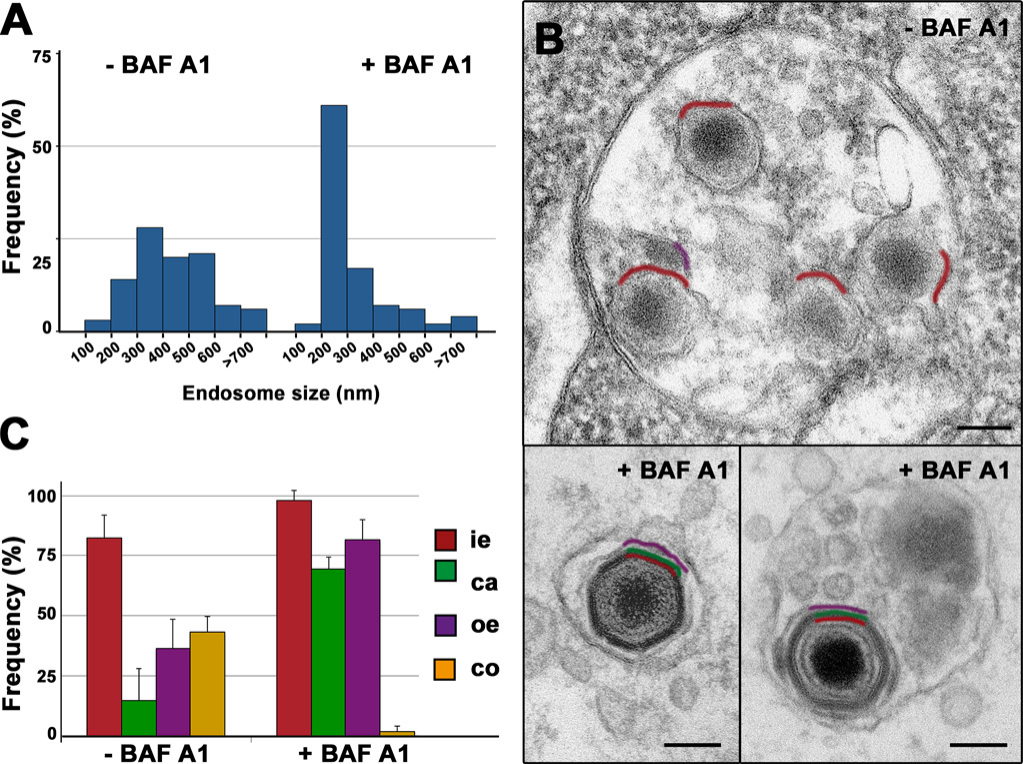
ASFV disassembly and uncoating depends on acidic pH. Swine macrophages were infected with ASFV for 90 min in the presence or absence of 100 nM Baf A1, an inhibitor of endosomal acidification, and then processed by EM. **A**) Size distribution of virus-containing endosomes (n>100) in the presence or absence of Baf A1. Note that after drug treatment, the virus-containing endosomes are significantly smaller as a consequence of the impairment of endosome maturation. **B**) Under these conditions, most ASFV particles appear essentially intact (lower panels). By contrast, most ASFV particles in non-treated cells undergo significant disruption at late endosomes (upper panel). Outer envelope (purple), capsid (green) and inner envelope (red) are indicated. Bars, 100 nm. **C**) Quantification of Baf A1 effect on virus disruption. Internalized particles from treated (+BAF A1) and nontreated (-BAF A1) cells were quantified according to their layer content (i.e: inner envelope, ca: capsid, oe: outer envelope, co: cytosolic core). Data are expressed as percentages of particles (mean ± SD of triplicate samples) with a given domain for each condition. More than 100 particles per experiment were analyzed. Note that Baf A1 significantly prevents ASFV disassembly and core release. Bars, 100 nm.

The inhibition of ASFV infection by Baf A1 and other inhibitors of endosome acidification [27, 29, 33, 34] strongly suggests that virus uncoating is pH dependent. To further investigate this possibility, we analyzed the effect of Baf A1 on the trafficking and ultrastructure of the incoming viruses in macrophages. The treatment with 100 nM Baf A1 for 90 min drastically altered the endocytic trafficking of the incoming virions. As shown in Fig 7A, the size distribution of the virus-containing vesicles in the presence (mean diameter: 340 ± 220 nm, n>100) or the absence (440 ± 160 nm, n>100) of the drug revealed a striking reduction after Baf A1 treatment, most likely as a consequence of a blockage in endosome maturation [40]. Most of the incoming particles were detected inside relatively small endocytic vesicles resembling early endosomes (Fig 7B, lower left panel). A minor fraction was, however, found inside larger, multivesicular endosomes (Fig 7B, lower right panel). Importantly, in both cases, the particles looked nearly intact (Fig 7B). In addition, essentially no cytosolic cores were detected. On the contrary, in control, non-treated infected cells, most of the incoming viruses were distributed between late endosomes and endolysosomal vesicles (Fig 7B, upper panel), showing evident signs of capsid disassembly as well as disruption of the outer envelope. EM quantification of virus layer content (Fig 7C) clearly evidenced that Baf A1 prevents both ASFV disruption and core release into the cytosol, which strongly suggests that these processes are pH-dependent.

**Fig 8:**
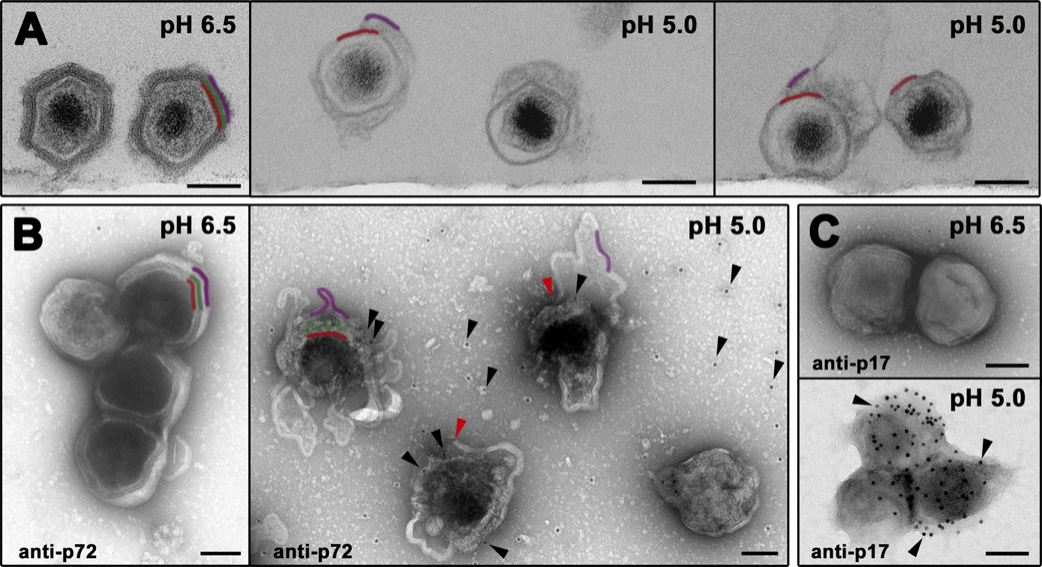
Low pH induces ASFV disruption. (**A**) *In vitro* effect of pH acidification on ASFV structure. Purified virions were exposed to pH 6.5 or 5.0 for 60 min at 37°C and then ultracentrifuged and analyzed by EM after ultrathin sectioning. Note that, at pH 6.5, particles look virtually intact while the particles exposed to acidic pH (5.0) are severely disrupted, displaying evident signals of capsid disassembly as well as disruption and detachment of the outer membrane. Outer envelope (purple), capsid (green) and inner envelope (red) are indicated. (**B**) Purified virions adsorbed to EM grids were exposed to pH 6.5 or 5.0 for 15 min at 37°C and then immunogold labeled for major capsid protein p72. Note that, at pH 6.5, particles look virtually intact remaining non-accessible for p72 labeling. In contrast, particles exposed to acidic pH (5.0) are severely disrupted. Under these conditions, gold-labeled anti-p72 antibodies (black arrowheads) penetrate the particles through the disrupted outer envelope (red arrowheads), decorating discrete regions where the capsid is still present. Also, p72 labeling is detected on the support film around the particles, as a consequence of capsid disassembly. (C) Purified virions adsorbed to EM grids were exposed to pH 6.5 or 5.0 for 15 min at 37°C and then labeled for major inner envelope protein p17. Note that particles disrupted at pH 5.0 become accessible to anti-p17 labeling. Bars, 100 nm.

On the basis of the above results, we explored the direct effect of pH acidification on the integrity of extracellular virions. To this end, purified particles were *in vitro* exposed to different balanced buffers ranging from pH 8.0 to 4.0. Most particles remained intact until pH dropped under 5.0, when they showed evident signals of disassembly. Next, virions were exposed to either pH 6.5 or 5.0 and analyzed by either ultrathin sectioning EM or immunogold staining for major capsid p72 protein and inner envelope protein p17. As shown in Fig 8A (left panel), particles exposed to pH 6.5 looked essentially intact, remaining inaccessible to anti-p72 (Fig 8B, left panel) or anti-p17 (Fig 8C, upper panel) labeling. In contrast, viruses exposed to pH 5.0 appeared severely disrupted displaying evident signals of capsid disassembly as well as outer membrane fragmentation and detachment (Fig 8A, right panels). Under these conditions, ASFV particles became accessible to anti-p72 antibodies (Fig 8B, right panel), which labeled discrete areas where the capsid components were still present. In addition, abundant p72 signal was detected on the support film, a further indication for capsid disassembly. Furthermore, the inner envelope of the disrupted particles became also accessible to anti-p17 antibodies (Fig 8C, lower panel). Altogether, *in vivo* and *in vitro* approaches indicate that pH acidification induces dissociation of the outer lipid membrane as well as capsid disassembly.

Finally, we analyzed if the viruses disrupted at pH 5.0 were infectious. To this, virus particles exposed to either pH 6.5 or 5.0 for 1h at 37°C were titrated in macrophages at 12 hpi by immunofluorescence with anti-p72 antibody. As shown in S8 Fig (left panel), acid-disassembled particles were ~ 4 fold less infectious than control intact particles exposed to pH 6.5. Interestingly, negative staining EM of the virus inoculum (S8 Fig, right panels) showed that virus particles exposed to acidic pH aggregate to a higher extent than control virions. This result would explain, at least in part, the observed drop of infectivity; however, it cannot be excluded that virus binding and entry are also affected.

### Core delivery is dependent on inner envelope protein pE248R

ASFV genome encodes a transmembrane protein, pE248R, which shares amino acid sequence similarity with VACV protein L1. Protein L1 is a component of the poxviral multiprotein entry/fusion complex, which is required for proper membrane fusion and/or core penetration [41, 42]. ASFV protein pE248R has been previously investigated by using an IPTG-dependent inducible recombinant [43]. Interestingly, in the absence of pE248R expression, virus morphogenesis and subsequent exit occur normally. However, the resulting viruses are noninfectious due to a blockage at an early post-entry stage prior to early viral transcription.

**Fig 9:**
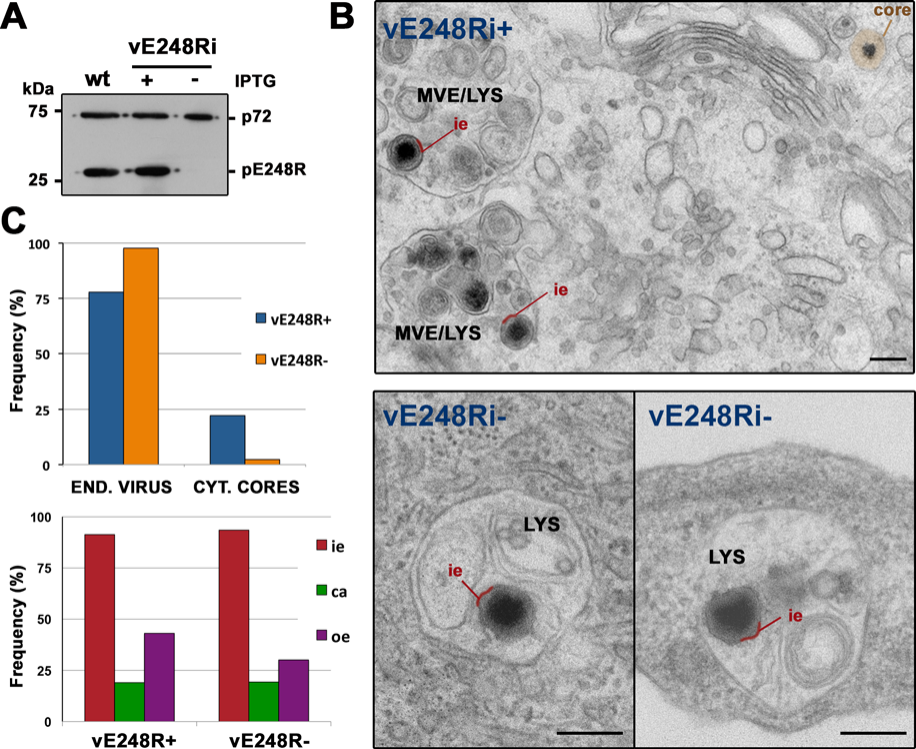
ASFV fusion depends on virus protein pE248R. **A)** Percoll-purified ASFV particles were obtained from cells infected with parental (wt) virus or recombinant virus vE248Ri under permissive (+IPTG) and non-permissive (-IPTG) conditions. Virus samples were analyzed by immunoblotting for the presence of capsid protein p72 and pE248R. **(B)** Vero cells were infected for 2 h with purified recombinant vE248Ri (~ 2 μg for 50,000 cells) grown with (+) or without (-) IPTG and then examined by EM. Disassembly of control vE248R+ (upper panel) and defective vE248R- (lower panels) was similar but core delivery into the cytosol was severely impaired for defective E248R- particles, which accumulated within lysosomes (LYS). To facilitate the interpretation, the inner viral envelope is depicted in red and the cytosolic viral core in brown. MVE (multivesicular endosomes). Bars, 200 nm. **C**) Quantification of disruption (lower panel) and core delivery (upper panel) of control E248R+ and defective E248R- particles. Endocytosed particles (END. VIRUS) and cytosolic cores (CYT. CORES) of the above experiment were quantified for both conditions and expressed as a percentage of intracellular virus. Also, the endocytosed particles of each condition were classified (lower panel) according to their layer content (i.e: inner envelope, ca: capsid, oe: outer envelope). One representative of two independent experiments is shown.

To further investigate the early role of pE248R, the internalization process of recombinant E248R-defective virus was examined in detail by EM. As a previous step, extracellular recombinant particles produced under permissive and restrictive conditions were purified by Percoll-density gradients. Western blot analysis confirmed the virtual absence of pE248R in the defective particles and the normal presence in those produced under permissive conditions (Fig 9A). Then, we compared the infection of Vero cells with equivalent amounts (according to protein estimation) of both kinds of virus particles. EM analysis at 2 hpi showed that internalization of recombinant ASFV particles containing pE248R protein was similar to that described before for the parental ASFV particles. A quantitative analysis revealed that about 22% of the intracellular particles were cytosolic cores, while about 78% were inside endocytic vesicles, mainly multivesicular endosomes and lysosomes (Fig 9B). At variance, in the infection with defective pE248R- particles (Fig 9C, upper panel), the proportion of cytosolic cores was drastically reduced (to less than 2.5%). The remaining intracellular particles (~ 97%) accumulated inside endocytic vesicles, many of them with a lysosome-like appearance (Fig 9B, lower panels). To ascertain if the diminished virus penetration was a consequence of altered uncoating, we analyzed the ultrastructure of endocytosed particles lacking pE248R protein. As shown in Fig 9C (lower panel), the disruption of defective pE248R- particles, as judged by the loss of the outer membrane and protein capsid, was similar to that of control pE248R+ particles. Altogether, these observations indicate that pE248R protein is not required for virus disassembly but for fusion and core delivery into the cytosol.

## Discussion

**Fig 10:**
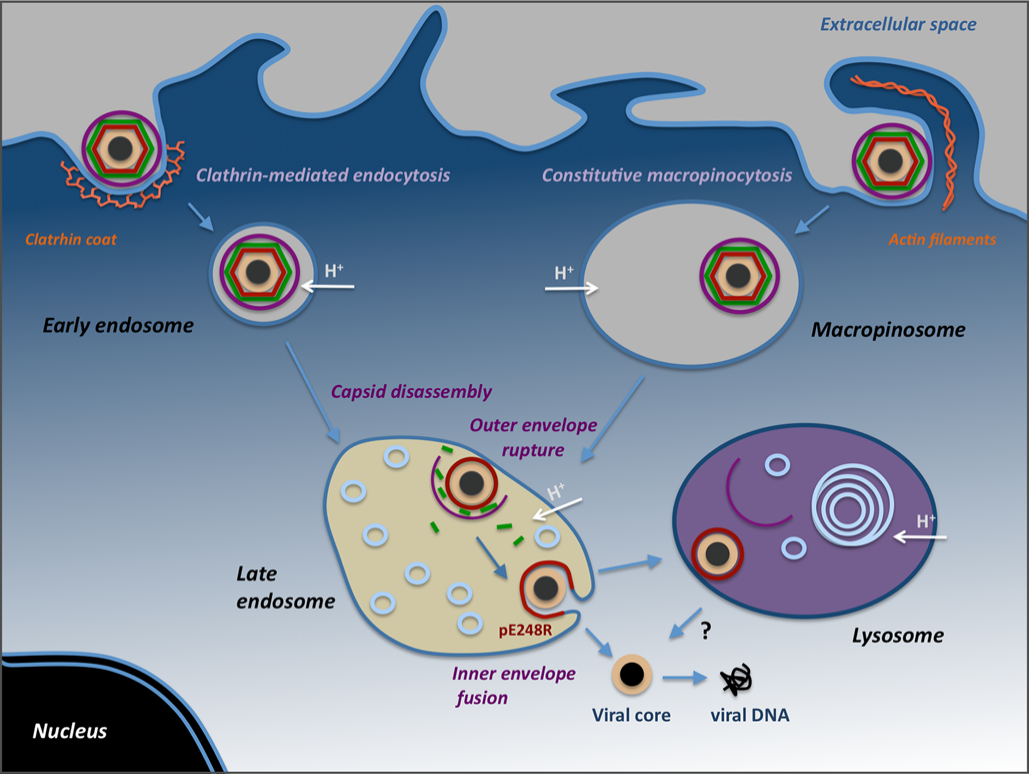
Model for ASFV internalization and uncoating. ASFV enters swine macrophages by clathrin-mediated endocytosis (left) and constitutive macropinocytosis (right). After the uptake, incoming particles are transported from early endosomes or macropinosomes to late endosomes, where they undergo the uncoating process, which would involve pH-dependent capsid disassembly and disruption of the outer viral membrane. Then, the exposed inner viral envelope fuses with the endosomal membrane to deliver genome-containing naked cores into the cytosol. A fraction of disrupted particles reach lysosomes, from where they might fuse or be further degraded. It cannot be excluded that lysosomal hydrolases (i.e. lipases and proteases) may also contribute to virus disruption or uncoating.

In this report, we have dissected the entry pathway of extracellular ASFV in swine macrophages, their target host cells. The results, summarized in Fig 10, indicate that ASFV can use two distinct endocytic pathways, clathrin-dependent endocytosis and macropinocytosis, to initiate a productive infection. Once endocytosed, ASFV particles become uncoated within multivesicular endosomes through a stepwise pH-dependent process leading to the loss of the outer lipid envelope and the protein capsid. The subsequent fusion of the inner envelope with the limiting endosomal membrane delivers naked cores into the cytosol.

ASFV entry and subsequent viral expression were strongly reduced by inhibitors of either CME (chloropromazine, pitstop2, and dynasore) or macropinocytosis (EIPA, IPA-3 and cytochalasin D). In keeping with these results, ASFV particles were visualized by EM inside clathrin coated pits and coated vesicles but also engulfed by plasma membrane protrusions into large cytoplasmic vesicles reminiscent of macropinosomes. The uptake of ASFV by macropinocytosis, which seems to represent the major entry route, is not surprising since it is a constitutive process in macrophages related with their continuous survey for extracellular foreign material and antigen presentation [9, 44]. The clathrin-dependent uptake is, in turn, more unexpected since it is usually employed by small and intermediate-sized viruses [3]. Coated vesicles are typically 50–100 nm in internal diameter due to the geometry imposed by the clathrin scaffold [7]. This constraint would theoretically preclude the uptake of large particles such as extracellular ASFV, with an average size of 200 nm. However, it has been shown that coated vesicles can be deformed to fit relatively large viruses like the bullet-shaped vesicular stomatitis virus (180x70 nm) [38, 45] or influenza A virus (~ 100 nm for the spherical form) [46]. According to our EM observations, this is also the case for extracellular ASFV particles, which are internalized within enlarged clathrin-coated vesicles with an average internal diameter of ~250 nm. ASFV is therefore one of the largest viruses internalized by clathrin-dependent endocytosis. The usage of multiple alternative endocytic mechanisms, such as CME and macropinocytosis, has been reported for a number of viruses including Ebola [47], Influenza A [48] or Singapore Grouper Iridovirus [49], a NCDLV structurally related to ASFV. Unlike macropinocytosis, CME is a selective, receptor-mediated endocytic mechanism. Early studies from our laboratory demonstrated that ASFV entry into macrophages occurs by receptor-mediated endocytosis, although nonsaturable uptake was also described [27, 28, 50]. Our present results are therefore consistent with such original findings. ASFV replicates mainly in macrophages and monocytes, although secondary target cells like vascular endothelial cells, hepatocytes or epithelial cells, have also been reported [51]. It is therefore conceivable that, by using different alternative pathways, ASFV increases its ability to adapt to the changing conditions of the infection process.

Our results clarify the highly contradictory evidence previously reported on the mechanism of ASFV entry. A recent study described that ASFV enters macrophages by CME, but not by macropinocytosis, despite the profound inhibitory effects of EIPA and cytochalasin D, together with various CME inhibitors, observed on early viral expression [30]. The mentioned report, however, did not provide direct evidence supporting or refuting each endocytosis mechanism. Another recent study stated that ASFV triggers its own internalization by macropinocytosis, but excluded the CME pathway [32]. Our results do not support such findings since classical cues of triggered macropinocytosis such as the induction of membrane ruffling, the activation of Pak 1 kinase or the increase of fluid-phase uptake, were not detected. A possible explanation for this contradictory evidence could be the use in the mentioned work of a clarified infection supernatant as virus inoculum. In our view, the presence of huge amounts of contaminant cell and viral debris [35], S1 Fig) might either elicit macropinocytosis and/or interfere with the interpretation of the results. A comparison of the scanning EM studies of both reports ([32], Fig 3 of this report) strongly supports this latter possibility.

Upon internalization, ASFV particles transit across the entire endocytic network [27, 33, 34] and this transport is paralleled with profound changes in the virus ultrastructure that critically depends on endosome maturation. Thus, incoming ASFV particles were first colocalized with early endocytic markers (TFR, EEA1, Rab5) and then with late endosome/lysosome markers (CD63, Rab7, Lamp1 and cathepsin L). Since classical endocytic markers are also present in the macropinosomes, these results are fully consistent with the usage by ASFV of both uptake pathways, which indeed converge at late endosomes and lysosomes [9].

Correlative light-electron microscopy and quantitative EM studies further demonstrated that virus disassembly occurs at Rab7+, multivesicular endosomes and subsequent endolysosomal structures. Consistent with this, ASFV infection in macrophages was prevented by pharmacological drugs interfering early endosome fusion (wortmannin), microtubule-dependent endosomal transport (nocodazole) and pH acidification (Baf A1). In addition, knockdown of Rab7a expression, a key regulator of late endosome maturation, blocked ASFV infection in COS-1 cells. Similar inhibitory effects have been reported in ASFV-infected Vero cells, although the ultrastructural details of virus uncoating were not analyzed [33]. Virus disruption involves a rapid disassembly of the protein capsid layer at multivesicular endosomes and a more gradual detachment of the outer lipid membrane, which reaches maximal levels at lysosomal-like vesicles displaying multilamellar content. These data suggest that the disruption of the outer membrane represents a rate-limiting step for subsequent fusion. Late endocytic vesicles probably provide the proper acidic pH environment [52] required for ASFV disassembly and fusion. In support of this, Baf A1 treatment prevented the virus disruption while the exposure of purified virions to acidic pH (5.0), which roughly corresponds to late endosomal pH, provoked the disruption of the outermost virus layers. Altogether, these findings suggest a mechanistic explanation to the previously reported inhibitory effects of lysosomotropic weak bases and v-ATPase inhibitors on ASFV infection [27, 29, 33, 34]. Thus, low pH could trigger conformational changes in the capsid structure leading to the dissociation of their building units and, consequently, to the detachment of the outer membrane. In relation to this, it is known that acidic pH may dissociate the capsid of some picornaviruses, like Foot-and-mouth disease virus, into their pentameric subunits [53]. This effect, which mimics the observed disassembly of the endocytosed virions, seems a consequence of the repulsive electrostatic interactions across the pentamer interface, which would display a high density of protonated histidine residues at acidic pH. In the case of ASFV, the molecular mechanism for low pH-induced destabilization of the capsid awaits a detailed elucidation of its structure.

The loss of the two outermost layers exposes the inner envelope, which then interacts and fuses with the limiting endosome membrane, as imaged by EM. Consistent with this, cytosolic naked cores, which are first detected at 45 min post-infection, appeared frequently in close proximity to multivesicular bodies and even lysosome-like vesicles. At this respect, it cannot be excluded that ASFV fusion may also occur at endolysosomes or lysosomes. Collectively, our data sustain the classification of ASFV as a late-penetrating virus [54]. It is conceivable that a simplified version of this mechanism would account for the internalization of the intracellular infectious form of ASFV, which lacks the outer membrane [26]. For both infectious ASFV forms, fusion at perinuclear late endosomes would allow the viral genome to approach to the region where viral DNA replication takes place.

In spite of the major differences in virion morphology and ultrastructure, the proposed model for ASFV entry is somewhat reminiscent of that described for the internalization of the extracellular infectious form of VACV, referred to as enveloped virion (EV). The brick-shaped EV particles consist of an inner core wrapped by two consecutive lipoprotein membranes. Unlike ASFV, poxviruses lack a proper protein capsid, although they form a transient capsid-like scaffold enwrapping the inner membrane during virus assembly [55, 56]. Upon macropinocytic uptake, EV particles lose their outer membrane by an acid-activated rupture process and then fuse their inner envelope with the limiting macropinosome membrane to release cytosolic cores [11, 12]. An uncoating process based on the disruption of the outer envelope and the fusion through the inner one might be a general mechanism shared by the multienveloped members of the NCLDV superfamily. In such case, the pathway of ASFV internalization would represent a more complex example involving an extra step of capsid disassembly.

Importantly, the analogies between the entry mechanisms of asfiviruses and poxviruses could be also extended to the fusion machinery. ASFV fusion depends upon viral protein pE248R, a myristoylated, type II transmembrane polypeptide located to the inner envelope [43]. Previous results showed that pE248R protein is required for a post-entry step of ASFV infection [43]. Here, we have shown that incoming ASFV particles defective in pE248R protein undergo normal disruption of the two outermost layers. However, they accumulate inside lysosome-like structures while the release of cores into the cytosol is severely abrogated. Strikingly, pE248R belongs to a viral cluster of myristoylated type II transmembrane polypeptides related to the VACV protein L1. This protein is a component of the poxviral multiprotein entry/fusion complex [57], which consists of, at least, 12 non-glycosylated, transmembrane proteins located at the inner viral membrane [41]. Studies with conditional lethal inducible mutants [42, 58] have shown that members of the entry/fusion complex, including L1 protein [41], are required for proper membrane fusion and/or core penetration. On the basis of these functional and structural similarities, it is tempting to speculate that protein pE248R is a component of a putative entry/fusion complex located at the ASFV inner membrane. In support of this hypothesis, another transmembrane ASFV protein, pE199L, belongs to a second cluster of viral proteins related with VACV proteins G9, A16 and J5, all of them also members of the entry/fusion complex. Interestingly, both viral groups include ORFs from different NCLDVs such as iridoviruses, phycodnaviruses, ascoviruses and mimiviruses (S9 Fig). Since NCLDVs are thought to share a common origin [59], it will be of great interest to address if the unconventional fusion machinery described in poxviruses represents an unifying concept extendible to the remaining NCLDVs.

In summary, our findings lead to a model for the internalization pathway of ASFV that could also explain the uncoating of other single and double-membraned icosahedral NCLDVs. In addition, this study provides new cellular and viral targets related with the first stages of ASFV infection, which could contribute to the development of novel antiviral strategies.

## Materials and Methods

### Cells

Porcine alveolar macrophages (maintained in our laboratory as a stock in liquid nitrogen) were obtained by lung lavage with phosphate-buffered saline (PBS) from healthy pigs, and maintained in Dulbecco's modified Eagle's medium (DMEM) supplemented with 10% heat inactivated swine serum, 2 mM L- glutamine, 100 U/ml gentamicin and nonessential amino acids. A basically pure culture of monocyte-macrophages was obtained by extensive washing of non-adherent cells after overnight culture. Vero and COS-1 cell lines, both from African green monkey kidney tissue, were obtained from the American Type Culture Collection and grown in DMEM containing 5% fetal bovine serum (FBS). All cell types were cultured at 37°C and 5% CO_2_ atmosphere.

### Viruses

All ASFV stocks used in this work were purified by two consecutive Percoll density gradients of clarified infection supernatants, as previously described [35]. The purity and integrity of virus preparations were monitored by SDS-PAGE and EM analysis after negative staining. BA71V, the Vero cell adapted ASFV isolate, and virulent ASFV isolate E70 were propagated in Vero or COS-1 cells, respectively. In general, BA71V virus was used in experiments with Vero and COS-1 cells whereas E70 virus was used to infect macrophages. For some assays related with virus transport and uncoating in macrophages, BA71V virus was also analyzed. Recombinant vE248Ri, a BA71V-derived recombinant virus with an inducible copy of the gene E248R [43], was propagated in Vero cells in the presence or absence of 0.25 mM isopropyl β-D-1-thiogalactopyranoside (IPTG) and purified by Percoll density gradients. VACV Western Reserve (WR) strain was kindly provided by Antonio Alcami (CBMSO, Madrid, Spain) and VACV expressing F13–GFP was a kind gift of Rafael Blasco (INIA, Madrid, Spain) [60]. Unless otherwise stated, viral infections were performed at 37°C after an adsorption period of 2 h at 4°C.

### Antibodies

The sources of the antibodies for the different markers were as follows: Rab7 (rabbit polyclonal), α-tubulin (mouse mAb, clone B-5-1-2) and β-Actin (mouse mAb; clone AC-15) were purchased from Sigma. EEA1 (rabbit mAb; clone C45B10,) and phospho-Pak-1-Thr423 (rabbit polyclonal) were from Cell Signaling Technologies. Total Pak-1 (rabbit polyclonal) was from Santa Cruz Biotechnology. CD63 (mouse mAb; clone H5C6) and Lamp-1 (mouse mAb; clone G1/139/5) were purchased from The Developmental Studies Hybridoma Bank (DSHB). Transferrin receptor (TRR, mouse mAb; clone H68.4) and GFP (rabbit polyclonal) were from Life Technologies. Clathrin heavy chain (CHC, mouse mAb; clone 23) was from BD Transduction Laboratories. Cathepsin L (rabbit polyclonal) was from Bioss. VACV 14K protein (mouse mAb) was a kind gift of Mariano Esteban (CNB, Madrid, Spain). The following antibodies against ASFV proteins were used in the present work: p17 (mouse mAb, clone 17K.G12, Ingenasa), p32 (affinity purified rabbit serum, [61]), p72 (clone 19B.A2, Ingenasa) and pE248R (rabbit serum, [43]).

Secondary antibodies conjugated to Alexa-488, -555, -594 or -647 were from Life Technologies. Direct labeling of anti-CD63 was performed with Zenon Alexa Fluor-555 mouse IgG1 labeling reagent (Life Technologies), following the manufacturer´s indications. Anti-rabbit Fab’ fragment coupled to 1.4 nm gold particles (Nanogold Antibody Conjugate, Cat No. 2004) and Gold enhancement (Cat No. 2113) were from NanoProbes. Horseradish peroxidase (HRP)-coupled secondary antibodies were from GE Healthcare.

### Drugs and reagents

The following pharmacological inhibitors were used: bafilomycin A1 (Sigma), cytochalasin D (Calbiochem), dynasore (Sigma), (5-(N-Ethyl-N-isopropyl) amiloride (EIPA, Sigma), 3-indolepropionic acid (IPA-3; Sigma), phorbol myristate acetate (PMA; Sigma), nocodazole (Sigma), pitstop2 (Abcam) and wortmaninn (Sigma). All of them were prepared as stock solutions in DMSO according to the manufacturer’s recommendation. Chlorpromazine (Sigma) was prepared in water just prior to use. Working solutions at indicated concentrations were freshly prepared in serum-free DMEM. Alexa Fluor 594-conjugated transferrin, Oregon Green-conjugated dextran (10 kDa), DiD, DAPI and Hoechst 33258 were from Life Technologies. FITC-labeled dextran (70 kDa) was from Sigma. Dextran-coated gold (30 nm) nanoparticles were from Nanocs.

### RNA interference

For RNA interference, all small interfering RNAs (siRNAs) were purchased from Sigma. To specifically interfere Rab7 expression, the following and previously described oligonucleotide sequences [62] were used: Rab7-1, sense sequence 5′-GGAUGACCUCUAGGAAGAATT; Rab7-2, sense sequence 5′-GAACACACGUAGGCCUUCATT. For knockdown of clathrin heavy chain (CHC), the previously described [63] oligonucleotide sequences were: CHC-1, sense sequence 5´-AAGCUGGGAAAACUCUUCAGA; CHC-2, sense sequence 5´-UAAUCCAAUUCGAAGACCAAU. As negative control we used the scrambled sequence: 5′-ACUUCGAGCGUGCAUGGCUTT-3′. COS-1 cells were seeded in 12-well plates to reach 40% confluence the day of transfection, while porcine macrophages were seeded directly to 80% confluence in 24-well plates two days before transfection. Cells were transfected with 60 pmoles of the corresponding siRNA using Lipofectamine 2000 (Life Technologies) in Opti-Mem medium (Life Technologies), according to the manufacturer´s recommendations. Knockdown was verified by immunoblotting 72 h after transfection.

### DNA constructs and transfection conditions

For transient expression assays, COS-1 cells were grown to 50% confluency and transfected with DNA constructs using Fugene-6 transfection reagent (Promega) and Opti-Mem (Life Technologies), following the manufacturer’s indications. Vectors encoding Rab5 and Rab7 GFP-tagged versions were kindly provided by J.A. Esteban (Centro de Biologia Molecular Severo Ochoa, Spain).

### DiD labeling of ASFV particles

Fluorescent DiD-labeled ASFV was obtained by incubating 25–50 μg of Percoll-purified particles (BA71V or E70 strains) with 1 μM of lipophilic dye DiD in PBS for 10 min at RT. Virus particles were sedimented at 20,000 g for 15 min, washed and resuspended into HBSS-Hepes (HBSS, 25 mM Hepes), snap frozen, and stored at −80°C.

### Flow cytometry-based virus entry assay

To measure ASFV uptake, macrophages were serum-starved for 12h. Then, the cells were incubated for 15 min with the indicated pharmacological inhibitors followed by infection with DiD-labeled ASFV particles (MOI 5) for 30 min at 37°C in the presence of the inhibitors. At this point, the cells were washed twice to remove unbound viruses and incubated for additional 30 min at 37°C in the presence of the inhibitors. Then, cells were incubated with trypsin-EDTA at 37°C to remove non-internalized virions and to detach cells, washed with PBS and fixed with 4% paraformaldehyde (PFA) for 30 min at 4°C. Finally, the cells were resuspended in FACS buffer (PBS, 0.01% sodium azide and 0.5% BSA and analyzed for DiD-emission (10^4^ cells/condition) in a FACSCalibur flow cytometer (BD Sciences). All FACS analyses were performed at least in triplicate and displayed as the mean fluorescence intensity normalized to control infection in the absence of a pharmacological inhibitor.

### Early viral expression analysis

To analyze the effect of entry inhibitors on early viral gene expression, serum starved cells were treated as above (i.e. 30 min of viral adsorption in the presence of inhibitors plus 30 min with only inhibitors) but the infection was extended for 2 additional hours (until 2.5 hpi) to allow the detection of early viral protein p32 by western immunoblotting as described below. This additional incubation was carried out in the absence of drugs to minimize possible post-entry inhibitory effects. The only exception was the treatment with dynasore, a reversible dynamin inhibitor, which was maintained along the infection.

### Western blot

Cell and virus samples were dissociated in 2X Laemmli buffer (2% SDS, 100 mM DTT, 125 mM Tris-HCl, pH 6.8), heated at 90°C for 5 min and electrophoresed on 12% polyacrylamide gels. Protein transfer was performed onto PVDF membranes (0.2 μm, Bio-Rad). Membranes were incubated with the following primary primaryantibodies at the indicated dilutions: anti-p32 (1:1000), anti-p72 (1:100), anti-pE248R (1:500), anti-CHC (1:1,000), anti-Rab7 (1:400), anti-Pak1 (1:500), anti-phospho Pak1 (1:500), anti-β-actin (1:5,000) and anti-α-tubulin (1:1,000). The anti-rabbit and anti-mouse secondary antibodies conjugated to HRP were used at a 1:10,000 dilution. Bands were developed with ECL Prime Western Blotting detection reagent (GE Healthcare), imaged with image analyzer ImageQuant LAS 4000 mini (GE Healthcare) and quantified with software package ImageQuant TL (GE Healthcare).

### Ruffling and blebbing assay

To analyze the induction of membrane blebbing, the protocol described in [12] was adapted. Briefly, Vero cells were grown on coverslips to 50% confluency, serum-starved for 6 h and incubated with DiD-labeled fluorescent ASFV particles (MOI 10) for 2h at 4°C. Then, infected cells were incubated for the indicated times until fixation. As positive controls, cells were infected with VACV (WR strain, MOI 10) or treated with 200 nM phorbol 12-myristate 13-acetate (PMA) for 15 min. After fixation with 4% PFA in PBS, cell nuclei and actin were detected with DAPI and Alexa Fluor-488 phalloidin, respectively. VACV particles were detected by immunofluorescence with a mouse mAb to VACV 14K protein. Fluorescence and differential interference contrast (DIC) images were recorded using a Leica DMI6000B automated inverted microscope equipped with a Hamamatsu Orca R2 digital camera.

### Fluid-phase uptake assay

Subconfluent Vero cells in 24-well plates were serum starved for 12 h before virus adsorption with ASFV or intracellular mature form of VACV (MOI 10) on ice for 60 min. After washing, cells were pulsed for 30 or 60 min with 0.5 mg/ml Oregon Green-conjugated 10-kDa dextran. As experimental control, cells were treated with 200 nM PMA at 37°C for 30 or 60 min min before the dextran pulse. Non-internalized dextran was removed by acid washing (0.1 M sodium acetate, 50 mM NaCl, pH 5.5) and cells were detached with trypsin/EDTA. After fixation in 4% PFA for 30 min at 4°C, cells were resuspended in 1% BSA, 1% FBS, 0.01% sodium azide in PBS. Dextran uptake was measured using a BD FACSCalibur flow cytometer (BD Sciences) and displayed as fluorescence mean of three independent experiments, normalized to mock-infected cell values.

### Immunofluorescence microscopy

Cells seeded on coverslips were serum starved for 12 h and infected for the indicated times, washed with PBS and fixed with 4% PFA in PBS for 15 min at RT. Then, cells were permeabilized with 0.2% saponin in PBS for 5 min at RT. After aldehyde quenching with 50 mM NH_4_Cl for 5 min and blocking with 10% FBS for 5 min, cells were incubated for 45 min at RT with primary antibodies at the following dilutions: anti-EEA1 (1:200), Zenon Alexa-555 labeled anti-CD63 (1:25), anti-p17 (1:500) and anti-p72 (19BA2, 1:100). Alexa-labeled secondary antibodies were diluted 1:500 and incubated for 45 min at RT. For triple labeling of endocytosed viruses, antibody incubation was as follows: anti-EEA1 together with anti-p17, then secondary antibodies Alexa Fluor-488 donkey anti-rabbit together with Alexa Fluor-647 goat anti-mouse and finally Zenon Alexa-555-labeled anti-CD63 antibody. All antibodies were diluted in 5% FBS in PBS. Cell nuclei were detected with DAPI (1 μg/ml) or Hoechst 33258 (5 μg/ml), and actin was detected with Alexa Fluor-488 phalloidin (1:100). Coverslips were mounted with ProLong Gold Antifade Mountant (Life Technologies) onto microscope slides. Images were recorded with a Leica DMI6000B automated inverted microscope equipped with a Hamamatsu Orca R2 digital camera or a Zeiss LSM510 Meta confocal system.

For co-internalization assays of virus, transferrin and dextran, serum starved macrophages were infected with ASFV particles (MOI 10) for 15 min at 37°C and then pulsed with Alexa fluor-488-labeled transferrin (100 μg/ml) and Alexa fluor-555 labeled 10-kDa dextran (0.5 mg/ml) for 10 additional min at 37°C. Cells were trypsinized for 2 min at 37°C and then washed three times with ice-cold PBS followed by cold acid washing for 5 min. Cells were then fixed for 30 min with 4% PFA, permeabilized with 0.1% saponin in PBS and processed for immunofluorescence with an antibody against capsid protein p72 (19BA2) followed by an Alexa 647-conjugated secondary anti-mouse antibody. For quantification, more than 250 colocalizing virus particles were counted.

### Transmission electron microscopy

For conventional EM, cells grown on carbon-coated 3-mm sapphire discs or on multiwell-24 plates were serum starved for 12 h and infected with 100–200 pfu/cell of ASFV (E70 or BA71V strains) in serum-free DMEM. At the indicated times, cells were *in situ* fixed with 4% PFA and 2% glutaraldehyde (GLA) in 0.1 M phosphate buffer (PB, pH 7.4) for 90 min at RT. Postfixation was carried out with 1% OsO_4_ and 1.5% K_3_Fe(CN)_6_ in water at 4°C for 1 h. Samples were dehydrated with acetone and *in situ* flat-embedded in Epoxy, TAAB 812 Resin (TAAB Laboratories) according to standard procedures. After polymerization, resin sheets containing the cell monolayers were detached from the substrate and mounted onto resin blocks to obtain orthogonal or parallel (from the bottom to the top of the cell) 80-nm ultrathin sections. The sections were deposited onto slot grids, stained with saturated uranyl acetate and lead citrate and examined at 80 kV in a Jeol JEM-1010 electron microscope. Images were recorded with a TemCam-F416 (4Kx4K) digital camera from TVIPS.

For dextran uptake assays, macrophages seeded on 3-mm sapphire discs were serum starved for 12 h and then incubated with virus (MOI 100) and/or dextran-coated 30-nm gold particles (final concentration: 5.0 x 10^11^ nanoparticles/ml) for 1 h at 4°C. Then, the samples were shifted to 37°C for 7 min. Cells were washed 4 times with PBS, fixed with 4% PFA and 2% GLA and processed for EM as above.

For quantification of ASFV-containing vesicles, ultrathin orthogonal sections were systematically screened at a magnification of 3,000–5,000X along a linear track from one end to the other of the EM slot grid. More than 100 vesicles within about 50–100 cell profiles were analyzed for every time point. The virus-containing endocytic vesicles were then analyzed at a magnification of 15,000–25,000X and classified using similar morphological criteria to those described in [64, 65]. Thus, the vesicles displaying an electron-lucent content, with none or a few internal vesicles (<5), were classified as primary endocytic vesicles and early endosomes/macropinosomes. These vesicles, which range from 200 to 500 nm (mean: 320 ± 105 nm, n>100), are predominant at 10 mpi. The dense endosomes with abundant intraluminal vesicles (>5) were classified as late endosomes/macropinosomes. The dense vacuoles containing electron-dense amorphous material and/or multilaminar membranes were classified as endolysosomes and lysosomes. These virus-containing late vesicles, which range from 300 to 800 nm (mean: 610 ± 185 nm, n>100), are predominant from 30 mpi onwards.

For quantification of virus disassembly and uncoating, a minimum of 100 endocytosed incoming viruses per condition (endocytic category or treatment) within about 50–100 cell profiles were analyzed at a magnification of 20,000–40,000X for the presence of the internal core, the inner membrane, the protein capsid and the outer membrane. When a given layer was incomplete (e.g. a broken, partially detached outer membrane), it was considered to be present only if covering more than 50% of the underlying particle. Data are then expressed as a percentage of endocytosed particles containing a given virus domain per condition.

To evaluate the effect of pH on ASFV ultrastructure, purified particles were exposed for 60 min at 37°C to 10 mM sodium citrate, pH 6.5 or 5.0, and 140 mM NaCl. Then, the particles were sedimented at 60,000 g for 10 min onto polylysine-coated, 3-mm Aclar plastic discs using a Beckman Airfuge equipped with an EM-90 rotor. Finally, samples were fixed with 4% PFA and 2% GLA and flat-embedded in epoxy resin before ultrathin sectioning in a plane orthogonal to the plastic disc.

### Immunoelectron microscopy

For immunoelectron microscopy, infected cells were *in situ* fixed with 2% PFA and 0.2% GLA or 4% PFA and 0.05% GLA in 0.1 M PB for 2 h at RT and kept in 1% (w/v) PFA in PB at 4°C. Subsequently, cells were embedded in 10% (w/v) gelatin and cryoprotected overnight in sucrose 2.3 M. Specimens were rapidly frozen in liquid nitrogen and cryosectioned with a Leica EM FCS cryo-ultramicrotome at -120°C. For immunogold labeling, thawed 90-nm thick cryosections were incubated with 20 mM glycine for 5 min at RT to quench free aldehyde groups and with 10% FBS for 5 min at RT to block nonspecific binding. Then, primary antibodies were incubated for 30 min at RT followed by protein A conjugated to 15-nm gold particles (EM Laboratory, Utrecht University, The Netherlands) or goat anti-mouse IgG conjugated to 15-nm gold particles (Aurion) for 30 min at RT. The primary antibodies and gold conjugates were diluted in PBS containing 5% FBS. The primary antibodies were used at the following dilutions: TFR (1:25), CD63 (1:50), Lamp1 (1:2) and Cathepsin L (1:250). Sections were stained with a mix of 1.8% methylcellulose and 0.4% uranyl acetate before visualization.

To evaluate the effect of pH on ASFV ultrastructure, purified particles were adsorbed to ionized collodion-carbon coated grids, washed with PBS and exposed for 15 min at 37°C to a drop of 10 mM sodium citrate, pH 6.5 or 5.0, and 140 mM NaCl. Then, grids were washed, fixed with 4% PFA for 5 min, and incubated with mAb anti-p72 (18BA2 clone) or mAb anti-p17 (17KG12) followed by protein A conjugated to 10-nm gold particles (EM Laboratory, Utrecht University, The Netherlands). Finally, specimens were negatively stained with 2% uranyl acetate before visualization.

### Correlative light-electron microscopy

An adaptation of the method described in [66] was employed. For *in vivo* fluorescence video microscopy, preconfluent COS-1 cells seeded onto gridded 35-mm glass-bottom dishes (MatTek) were transfected with plasmids encoding Rab5-gfp or Rab7-gfp as above described. One day after transfection, cells were infected with DiD-labeled ASFV particles (MOI 25) in serum-free DMEM containing Hoechst 33258 (1 μg/ml). After a 30-min adsorption period at 4°C, time-lapse microscopy of selected ASFV-infected, Rab5- or Rab7-transfected cells was performed with a Leica DMI6000B automated inverted microscope equipped with a Hamamatsu Orca R2 digital camera. Following multi-channel time-lapse fluorescence and DIC imaging, cells were fixed with 4% PFA and 0.05% GLA in PBS for 30 min at RT. For immunolabeling, fixed cells were incubated for 30 min with a blocking/permeabilizing (B/P) solution (0.5% BSA, 0.1% saponin, 50 mM NH_4_Cl) and subsequently overnight at 4°C with anti-GFP antibodies, diluted 1:1000 in B/P solution. After extensive washing with PBS, cells were incubated with the anti-rabbit Fab’ fragment coupled to 1.4 nm gold particles (Nanoprobes) diluted 1:100 in B/P solution) for 2 h at RT. A gold-enhancement reaction (Nanoprobes) was performed during 5–10 min to increase the size of the 1.4 nm gold particles. Following immunolabeling, cells were postfixed for 1 h on ice with 1% OsO_4_ and 1.5% K_3_Fe(CN)_6_ and then overnight at 4°C with 0.5% uranyl acetate. Finally, cells were dehydrated with ethanol and flat-embedded in Epoxy, TAAB 812 Resin. After polymerization and resin detaching, selected cells were identified at the optical microscope with the help of the coordinated grid. Ultrathin serial sections (from basal to apical) were obtained in an orientation parallel to the cell plane. Sections were collected onto zinc slot grids and visualized at the transmission electron microscope to detect ASFV particles inside immunogold-labeled endocytic vesicles. To this purpose, the previously acquired DIC and fluorescence images were used to identify and correlate the areas of interest.

### Scanning electron microscopy

For scanning EM, cells were seeded on 7-mm glass coverslips, serum starved for 12 h and infected with ASFV particles (MOI 100 or ~10,000 physical particles/cell) for 20 min at 37°C. Then, cells were fixed in 4% (w/V) PFA and 2% (w/V) GTA in 0.1 M PB (pH 7.4) for 2 h at RT and postfixed in 2% OsO_4_ water at 4°C for 60 min. Samples were dehydrated in ethanol, critical point dried for 2 h and coated with graphite-gold in a sputter coater. Finally, specimens were analyzed with a Jeol JSM-6335-F field emission scanning electron microscope (Electron Microscopy National Center, UCM, Madrid, Spain) operating at 5 kV.

### Statistical analysis

Unless otherwise indicated, the data are representative of at least three independent experiments, and values are given as the mean of triplicates ± standard deviation (SD).

## Supporting Information

S1 Fig EM evaluation of ASFV purification. Infection supernatants were subjected to low-speed centrifugation to remove most cell debris and then to high-speed sedimentation as described [32]. The virus preparation (**A**) was then centrifuged through a 40% sucrose cushion (**B**) as described in [30] or subjected to two consecutive Percoll density gradients followed by a size-exclusion chromatography (**C**). Note that while the virus preparations A and B exhibit huge amount of membrane and particulate debris (black arrows), the Percoll-based method used in this report produces virtually homogeneous ASFV particles. The arrows in C indicate Percoll beads. **D-E**) Vero cells were incubated for 2 h at 4°C with ASFV particles (MOI 50) semipurified through 40% sucrose cushion (**D**) or by Percoll-density gradient (**E**) and then at 37°C for 30 min. After extensive washing, cells were fixed and processed by EM. Note the significant presence of attached cell debris (arrows) in D. ASFV particles (red arrowheads) are indicated. Bars, 500 nm.
(TIF)

S2 Fig Effect of CME inhibitors on ASFV and VACV infection. Macrophages pre-treated for 15 min with the CME inhibitors CPZ (15 μM), PTS2 12 μM, DYN (100 μM) and sucrose (0.45M) were infected for 1h at 37°C with ASFV or a recombinant VACV expressing F13L-gfp gene, in the presence of the inhibitors (except for hyperosmotic sucrose). Then, the cells were washed to remove inhibitors and unbound virus and incubated for 12 h at 37°C. After fixation, ASFV-infected cells were labeled for immunofluorescence with an antibody against capsid protein p72. VACV-infected cells were directly detected by the expression of fluorescent F13L-gfp protein. Data are expressed as percentage of infected cells to a control infection (mean of two independent experiments ± SD.
(TIF)

S3 Fig CME and macropinocytosis explain most of ASFV entry in macrophages. **(A)** Macrophages pre-treated for 15 min with 15 μM CPZ, 40 μM EIPA or a combination of them were incubated with DiD-labeled fluorescent ASFV particles (MOI 5) for 30 min. Then, the cells were incubated for an additional 30 min period in the presence of inhibitors and analyzed for virus uptake by flow cytometry. Data are expressed as relative fluorescence to a control infection (mean of six independent experiment ± SE). (**B)** In a second set of experiments, macrophages were treated as above but infection was extended to 2.5 hpi to allow detection of the expression of early viral protein p32 by immunoblotting.
(TIF)

S4 Fig ASFV entry by clathrin-mediated endocytosis. Vero cells were infected with ASFV (MOI 50) for 30 min at 37°C after a 2-h adsorption at 4°C. Then, cells were processed either by conventional epon embedding (**A-D**) or by cryosectioning (**E-G**). Thawed cryosections were incubated with a mouse antibody against clathrin heavy chain followed by protein A-gold (10 nm) conjugates. Note virions at coated pits (A,B, C, E, F) and coated vesicles (D and G). Clathrin coats (arrows) and immunogold labeling (arrowheads) are indicated. Bars, 100 nm.
(TIF)

S5 Fig Field Emission Scanning EM of Mock- and ASFV-infected Vero cells at 30 mpi (MOI 200). Virus particles of central panel are depicted in blue. Red lines in the right panel indicate cell boundaries. Bars, 1 μm.
(TIF)

S6 Fig Correlative light-electron microscopy of ASFV transit. COS-1 cells expressing Rab5-gfp (A-E) or Rab7-gfp (F-I) were incubated with DiD-labeled ASFV particles (MOI 25) for 30 min at 4°C and then for 15 (Rab5-gfp) or 30 min (Rab7-gfp) at 37°C. Selected cells were analyzed by time-lapse fluorescence and DIC microscopy (**A** and **F**; see also S4 Video for Rab7-expressing cells). After fixation and saponin permeabilization, cells were incubated with a rabbit anti-GFP antibody followed by an anti-rabbit Fab´ conjugated to 1.4-nm gold nanoparticle. Then, the GFP signal was amplified by gold enhancement (**B**) and the cells were postfixed, flat-embedded and serial sectioned from the basal to the apical side (**C**). Finally, selected cells were analyzed at the EM level for the presence of endocytosed ASFV particles. As an example, panel **D** shows an EM section of the cell expressing Rab5-gfp shown in panel A. Panels **E** show EM micrographs of virus particles inside Rab5+ endosomes, which correspond to those identified by numbers (1 to 4) in the fluorescence image (**A**). The same procedure was followed for Rab7-gfp transfected cells (F-I). Panel **H** (and lower inset) shows two virus particles inside a Rab7+ late endosome (identified as 2 in panel F) whose movement was recorded by time-lapse microscopy (S4 Video). Panel **I** (and lower inset) shows a virus particle inside a Rab7+ endolysosome-like structure (number 3). As reference, panel **G** shows a nearly intact virus inside a putative early endosome (number 1) of a neighbor, non-transfected cell. Panel G also illustrates the background level of the immunolabeling procedure. Note that the virus particles inside Rab5+ vesicles (E) look nearly intact and display icosahedral morphology whereas those particles inside Rab7+ vesicles look disrupted. Bars, 2 μm (A, D and F), 500 nm (G, H, I), 100 nm (E and lower insets of G, H, I).
(TIF)

S7 Fig Endocytic transport and uncoating of ASFV in Vero cells. Virus-infected cells (MOI 200) were fixed and processed by EM at 10, 30, 45, 60 and 120 min. **A-D**) After endocytosis, incoming particles are detected at 10 mpi within relatively small endocytic vesicles, where they look nearly intact (**A**, **B** and **C**). A small proportion of particles show signs of capsid disassembly (**D**). **E-H**) At 30–60 mpi, particles are preferentially detected within large multivesicular endosomes (MVE). At this stage, most of the particles (80%) lack the protein capsid (**E**, **F** and **G**) and a significant proportion (50%) lack the outer envelope (**G** and **H**) or it appears partially dissociated (**F**). **I**) At later times (120 mpi), most endocytic particles are inside lysosome-like structures (LYS) and look completely uncoated. Released virus cores (**H**, **I**, and **J**), which consist of a dense nucleoid (nu) wrapped by a thick core shell (cs) and a limiting thin core coat (cc), can be detected from 60 mpi onwards in close proximity to multivesicular (**H**) and/or multilamellar (**I**) endosomes. Bars, 200 nm.
(TIF)

S8 Fig Acid-disassembled ASFV particles are less infectious. Purified ASFV particles were exposed to either pH 6.5 or 5.0 for 1h at 37°C. After pH neutralization, virus particles were titrated by triplicate in macrophages at 12 hpi by immunofluorescence with anti-p72 antibody (left panels). Bars, 15 μm. ASFV particles disrupted at pH 5.0 are ~ 4 fold less infectious than control intact particles exposed to pH 6.5. Control and acid-treated virus particles (n>300) were also analyzed and quantified for aggregation by negative staining EM (right panels). The histograms show the frequency of particles for each aggregation class/category. Note that acid-treated virions aggregate to a higher extent (48% of virions were present as double-particle or higher aggregates) than control virions (15%). Bars, 200 nm.
(TIFF)

S9 Fig ASFV protein pE248R belongs to a viral cluster related to VACV protein L1. Amino acid sequence alignment of some viral orthologs of VACV protein L1, a member of the entry/fusion complex. The protein cluster (pfam02442: L1R_F9L) includes putative and known transmembrane polypeptides of several NCLDV families like Poxviridae (L1R), Asfarviridae (pE248R), Iridoviridae, Ascoviridae and Mimiviridae. Some selected members of the cluster are shown (complete sequence alignment can be found at http://www.ncbi.nlm.nih.gov/Structure/cdd/cddsrv.cgi?uid=pfam02442&islf=1). Amino acids exhibiting some degree of conservation are shown in upper case, and red color indicates highly conserved residues among rows (default 2.0-bit threshold is shown) while blue indicates less conserved residues.
(TIFF)

S1 Video Colocalization of DiD-labeled ASFV with Rab5+ endosomes. Transfected COS-1 cells expressing Rab5-gfp (green) were infected with DiD-labeled ASFV particles (red) and analyzed by time-lapse fluorescence at 10–20 mpi. The blue arrowheads indicate transport of ASFV particles inside Rab5+ endosomes. Bar, 10 μm.
(AVI)

S2 Video Colocalization of DiD-labeled ASFV with Rab5+ endosomes. Transfected COS-1 cells expressing Rab5-gfp (green) were infected with DiD-labeled ASFV particles (red) and analyzed by time-lapse fluorescence at 10–20 mpi. The blue arrowhead indicate transport of an ASFV particle inside a Rab5+ endosome. Bar, 5 μm.
(AVI)

S3 Video Colocalization of DiD-labeled ASFV with Rab7+ endosomes. Transfected COS-1 cells expressing Rab7-gfp (green) were infected with DiD-labeled ASFV particles (red) and analyzed by time-lapse fluorescence at 30 mpi. The blue arrowheads indicate ASFV paricles inside Rab7+ endosomes. Bar, 10 μm.
(AVI)

S4 Video Colocalization of DiD-labeled ASFV with Rab7+ endosomes. Transfected COS-1 cells expressing Rab7-gfp were infected with DiD-labeled ASFV particles and analyzed by time-lapse fluorescence at 30 mpi. The video shows the movement of incoming ASFV particles (red) inside Rab7+ endosomes (green). At the end of the time-lapse capture, cells were fixed and processed for correlative light-electron microscopy as shown in S5 Fig. The blue arrowhead indicates a virus-containing late endosome (number 2) analyzed at the EM level in S5 Fig. Bar, 5 μm.
(AVI)
