# Supplementary figures and images for: African Swine Fever Virus Undergoes Outer Envelope Disruption, Capsid Disassembly and Inner Envelope Fusion before Core Release from Multivesicular Endosomes
Source: PLoS Pathog. 2016 Apr 25;12(4):e1005595. doi: 10.1371/journal.ppat.1005595 (PMC4844166; doi:10.1371/journal.ppat.1005595)

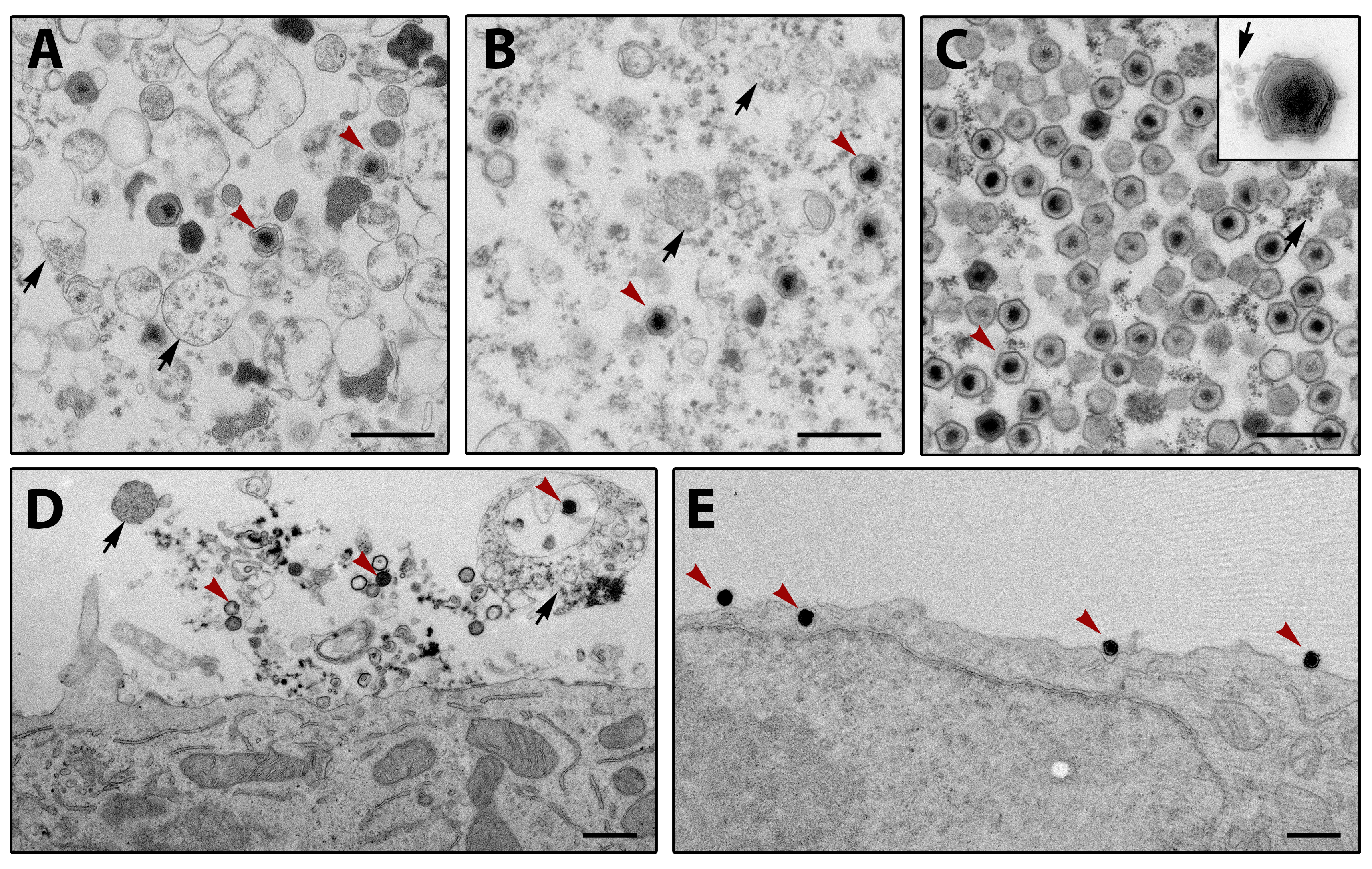

Supplement: S1 Fig — Infection supernatants were subjected to low-speed centrifugation to remove most cell debris and then to high-speed sedimentation as described [32]. The virus preparation (A) was then centrifuged through a 40% sucrose cushion (B) as described in [30] or subjected to two consecutive Percoll density gradients followed by a size-exclusion chromatography (C). Note that while the virus preparations A and B exhibit huge amount of membrane and particulate debris (black arrows), the Percoll-based method used in this report produces virtually homogeneous ASFV particles. The arrows in C indicate Percoll beads. D-E) Vero cells were incubated for 2 h at 4°C with ASFV particles (MOI 50) semipurified through 40% sucrose cushion (D) or by Percoll-density gradient (E) and then at 37°C for 30 min. After extensive washing, cells were fixed and processed by EM. Note the significant presence of attached cell debris (arrows) in D. ASFV particles (red arrowheads) are indicated. Bars, 500 nm. (TIF) [file ppat.1005595.s001.tif]

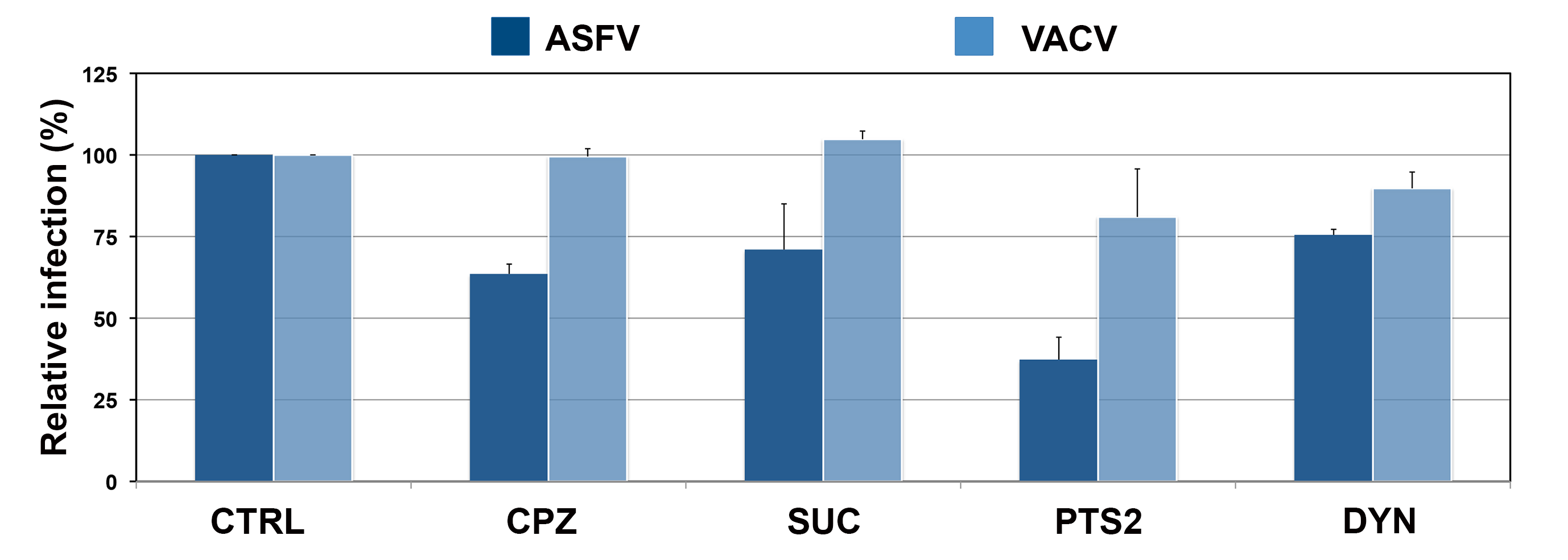

Supplement: S2 Fig — Macrophages pre-treated for 15 min with the CME inhibitors CPZ (15 μM), PTS2 12 μM, DYN (100 μM) and sucrose (0.45M) were infected for 1h at 37°C with ASFV or a recombinant VACV expressing F13L-gfp gene, in the presence of the inhibitors (except for hyperosmotic sucrose). Then, the cells were washed to remove inhibitors and unbound virus and incubated for 12 h at 37°C. After fixation, ASFV-infected cells were labeled for immunofluorescence with an antibody against capsid protein p72. VACV-infected cells were directly detected by the expression of fluorescent F13L-gfp protein. Data are expressed as percentage of infected cells to a control infection (mean of two independent experiments ± SD. (TIF) [file ppat.1005595.s002.tif]

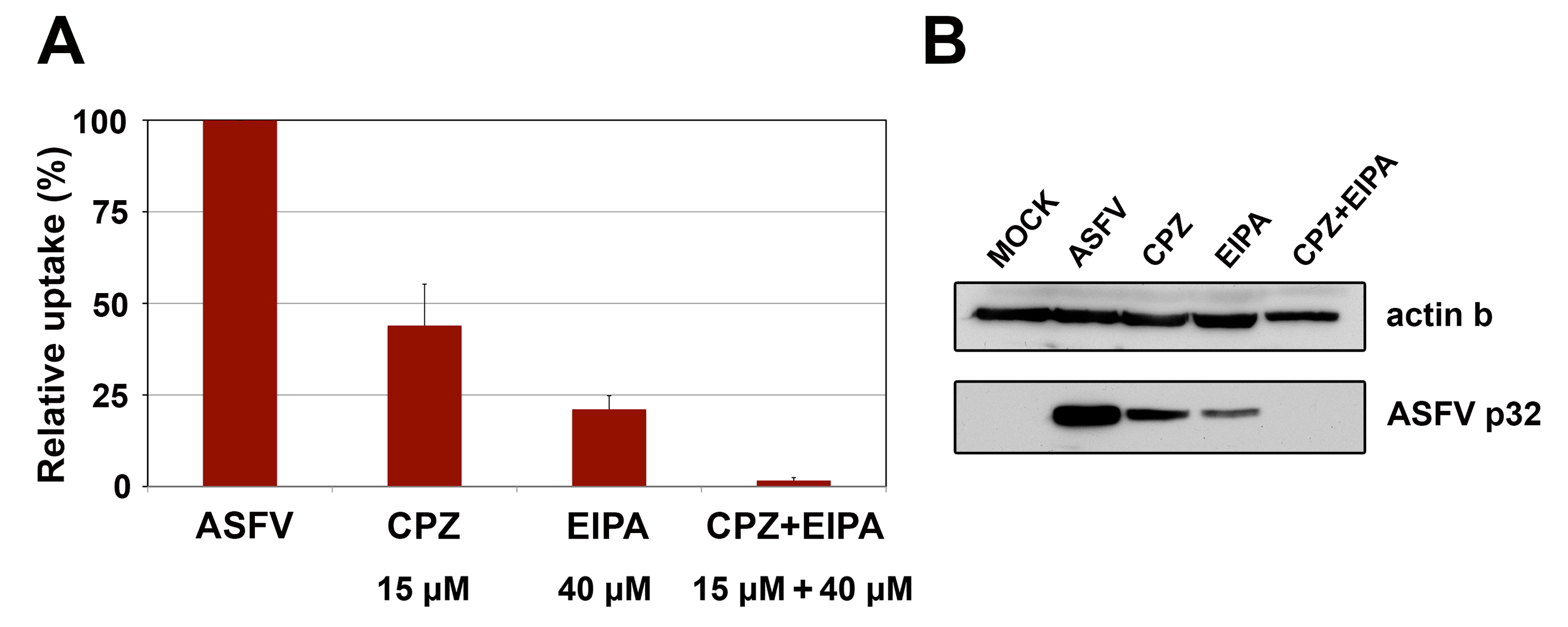

Supplement: S3 Fig — (A) Macrophages pre-treated for 15 min with 15 μM CPZ, 40 μM EIPA or a combination of them were incubated with DiD-labeled fluorescent ASFV particles (MOI 5) for 30 min. Then, the cells were incubated for an additional 30 min period in the presence of inhibitors and analyzed for virus uptake by flow cytometry. Data are expressed as relative fluorescence to a control infection (mean of six independent experiment ± SE). (B) In a second set of experiments, macrophages were treated as above but infection was extended to 2.5 hpi to allow detection of the expression of early viral protein p32 by immunoblotting. (TIF) [file ppat.1005595.s003.tif]

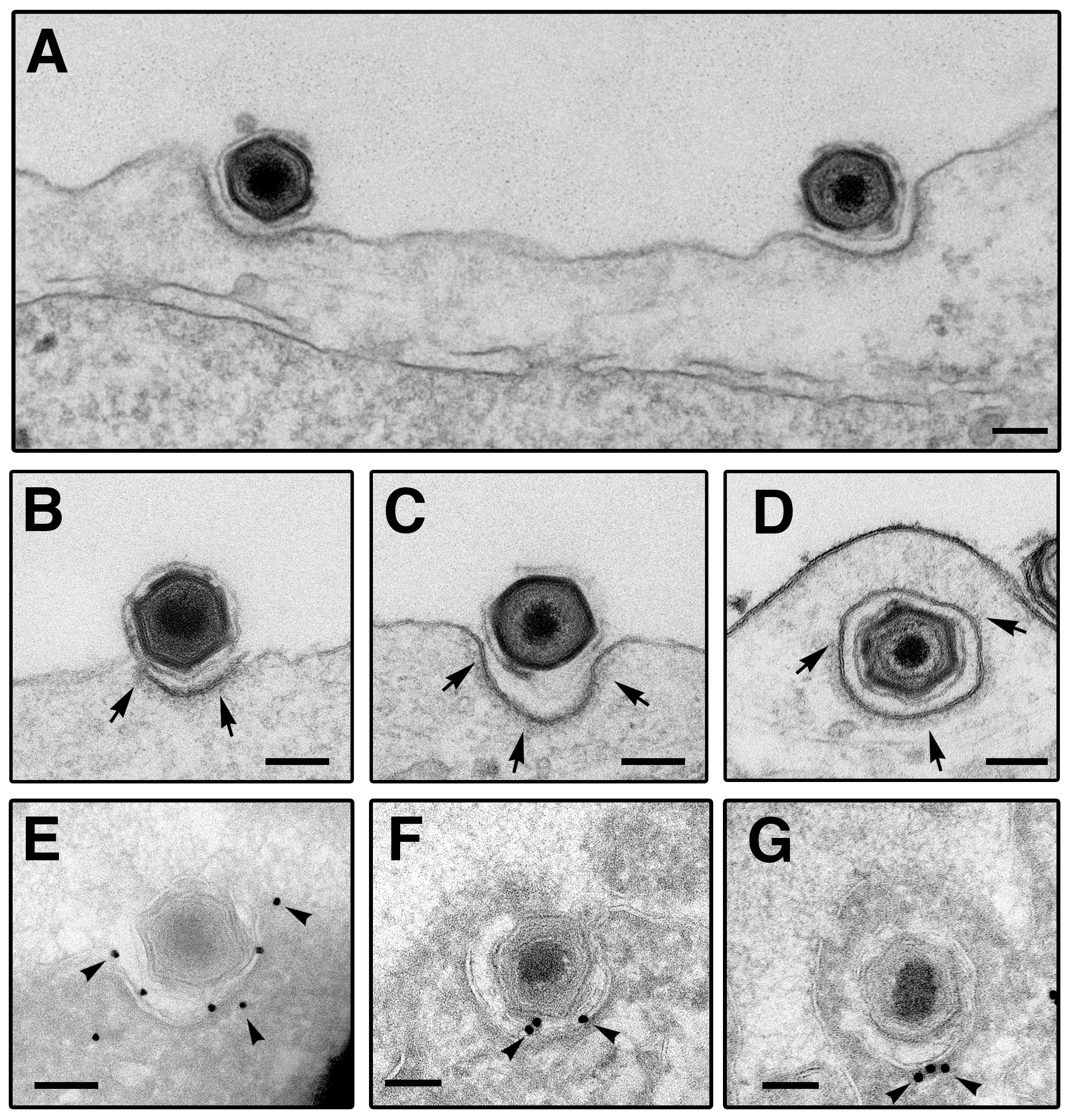

Supplement: S4 Fig — Vero cells were infected with ASFV (MOI 50) for 30 min at 37°C after a 2-h adsorption at 4°C. Then, cells were processed either by conventional epon embedding (A-D) or by cryosectioning (E-G). Thawed cryosections were incubated with a mouse antibody against clathrin heavy chain followed by protein A-gold (10 nm) conjugates. Note virions at coated pits (A,B, C, E, F) and coated vesicles (D and G). Clathrin coats (arrows) and immunogold labeling (arrowheads) are indicated. Bars, 100 nm. (TIF) [file ppat.1005595.s004.tif]

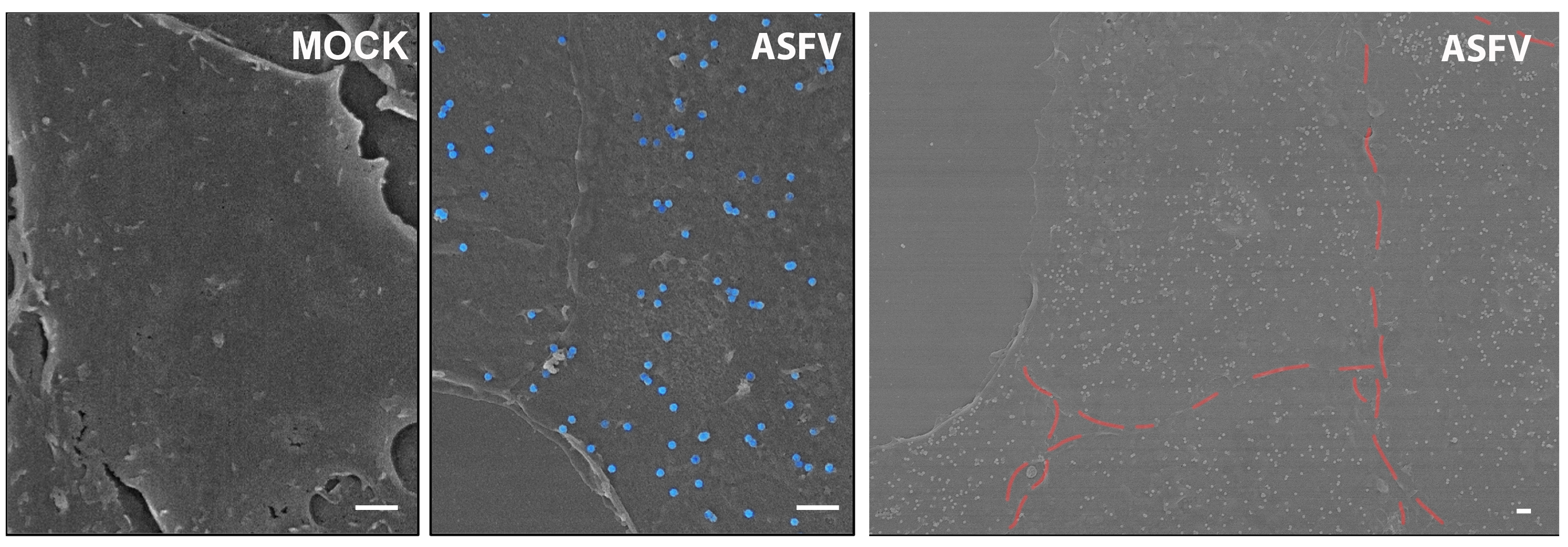

Supplement: S5 Fig — Virus particles of central panel are depicted in blue. Red lines in the right panel indicate cell boundaries. Bars, 1 μm. (TIF) [file ppat.1005595.s005.tif]

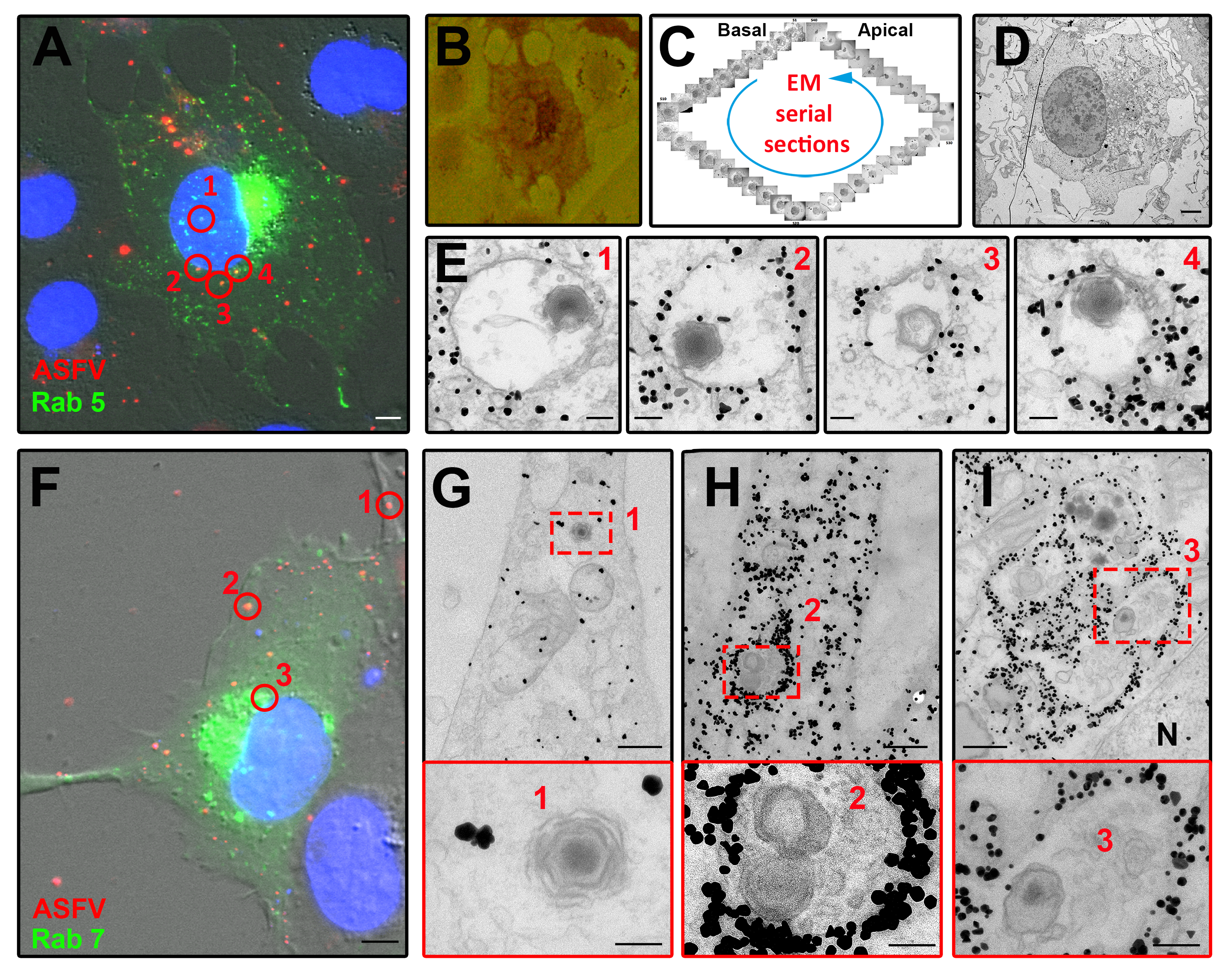

Supplement: S6 Fig — COS-1 cells expressing Rab5-gfp (A-E) or Rab7-gfp (F-I) were incubated with DiD-labeled ASFV particles (MOI 25) for 30 min at 4°C and then for 15 (Rab5-gfp) or 30 min (Rab7-gfp) at 37°C. Selected cells were analyzed by time-lapse fluorescence and DIC microscopy (A and F; see also S4 Video for Rab7-expressing cells). After fixation and saponin permeabilization, cells were incubated with a rabbit anti-GFP antibody followed by an anti-rabbit Fab´ conjugated to 1.4-nm gold nanoparticle. Then, the GFP signal was amplified by gold enhancement (B) and the cells were postfixed, flat-embedded and serial sectioned from the basal to the apical side (C). Finally, selected cells were analyzed at the EM level for the presence of endocytosed ASFV particles. As an example, panel D shows an EM section of the cell expressing Rab5-gfp shown in panel A. Panels E show EM micrographs of virus particles inside Rab5+ endosomes, which correspond to those identified by numbers (1 to 4) in the fluorescence image (A). The same procedure was followed for Rab7-gfp transfected cells (F-I). Panel H (and lower inset) shows two virus particles inside a Rab7+ late endosome (identified as 2 in panel F) whose movement was recorded by time-lapse microscopy (S4 Video). Panel I (and lower inset) shows a virus particle inside a Rab7+ endolysosome-like structure (number 3). As reference, panel G shows a nearly intact virus inside a putative early endosome (number 1) of a neighbor, non-transfected cell. Panel G also illustrates the background level of the immunolabeling procedure. Note that the virus particles inside Rab5+ vesicles (E) look nearly intact and display icosahedral morphology whereas those particles inside Rab7+ vesicles look disrupted. Bars, 2 μm (A, D and F), 500 nm (G, H, I), 100 nm (E and lower insets of G, H, I). (TIF) [file ppat.1005595.s006.tif]

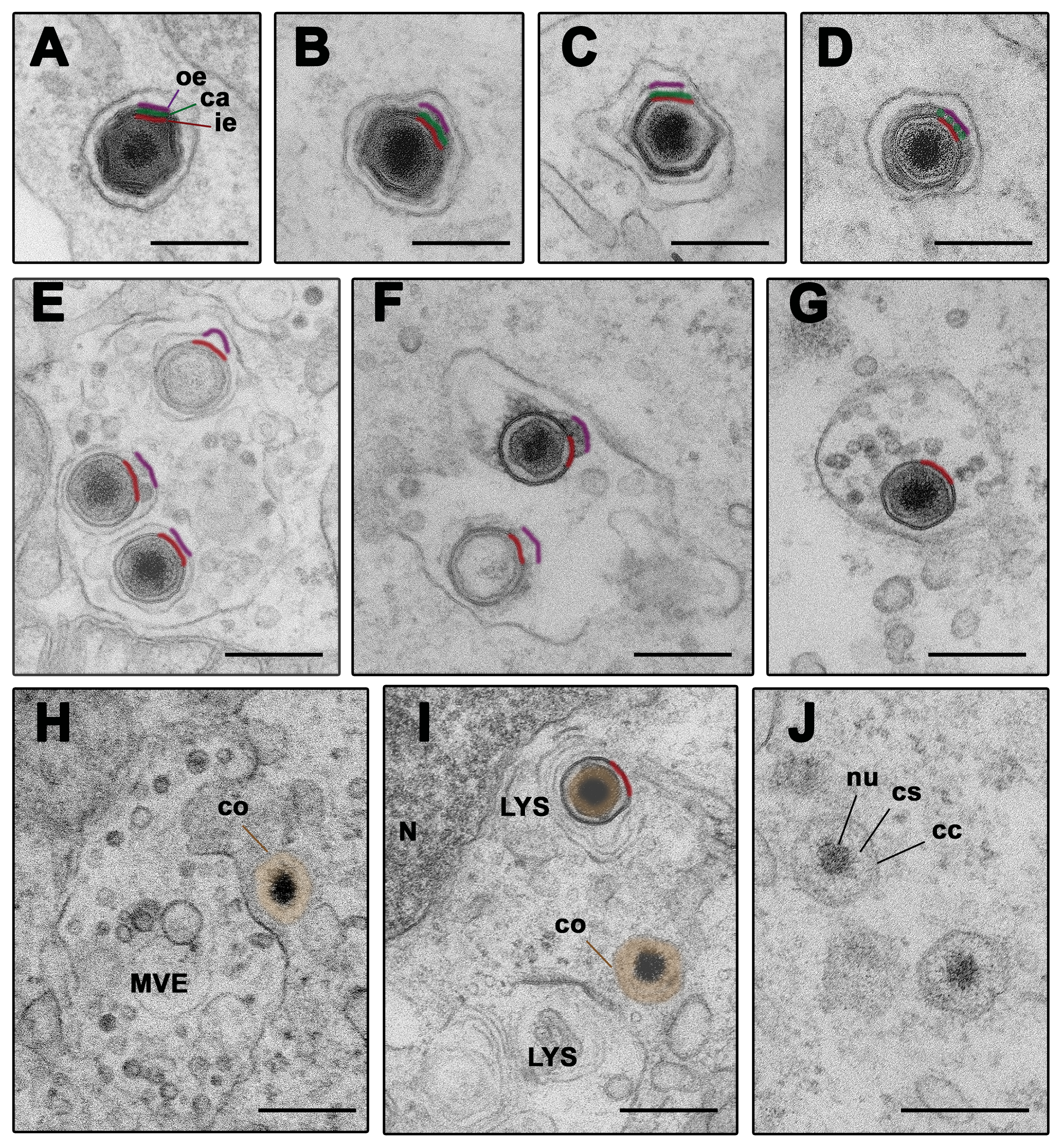

Supplement: S7 Fig — Virus-infected cells (MOI 200) were fixed and processed by EM at 10, 30, 45, 60 and 120 min. A-D) After endocytosis, incoming particles are detected at 10 mpi within relatively small endocytic vesicles, where they look nearly intact (A, B and C). A small proportion of particles show signs of capsid disassembly (D). E-H) At 30–60 mpi, particles are preferentially detected within large multivesicular endosomes (MVE). At this stage, most of the particles (80%) lack the protein capsid (E, F and G) and a significant proportion (50%) lack the outer envelope (G and H) or it appears partially dissociated (F). I) At later times (120 mpi), most endocytic particles are inside lysosome-like structures (LYS) and look completely uncoated. Released virus cores (H, I, and J), which consist of a dense nucleoid (nu) wrapped by a thick core shell (cs) and a limiting thin core coat (cc), can be detected from 60 mpi onwards in close proximity to multivesicular (H) and/or multilamellar (I) endosomes. Bars, 200 nm. (TIF) [file ppat.1005595.s007.tif]

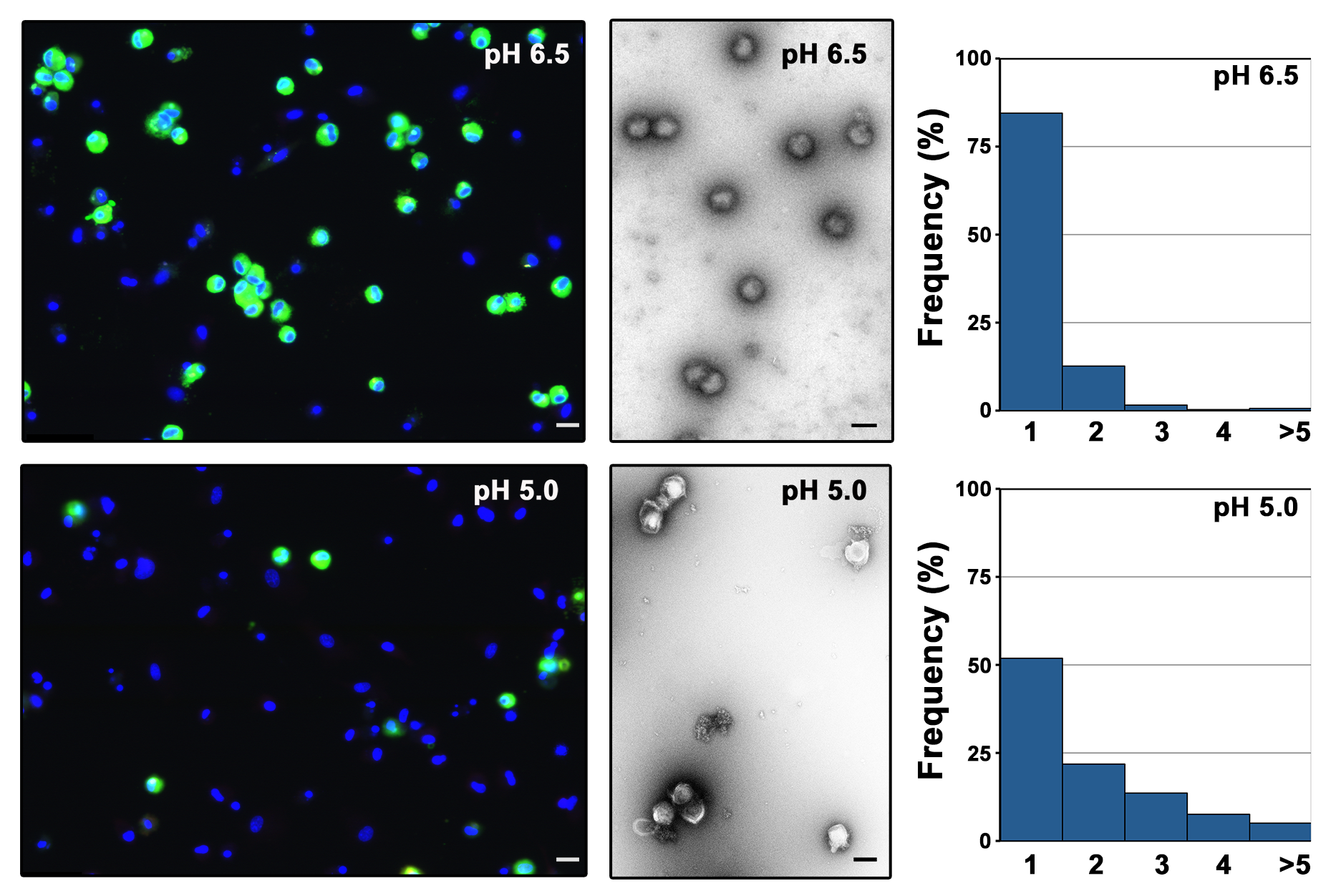

Supplement: S8 Fig — Purified ASFV particles were exposed to either pH 6.5 or 5.0 for 1h at 37°C. After pH neutralization, virus particles were titrated by triplicate in macrophages at 12 hpi by immunofluorescence with anti-p72 antibody (left panels). Bars, 15 μm. ASFV particles disrupted at pH 5.0 are ~ 4 fold less infectious than control intact particles exposed to pH 6.5. Control and acid-treated virus particles (n>300) were also analyzed and quantified for aggregation by negative staining EM (right panels). The histograms show the frequency of particles for each aggregation class/category. Note that acid-treated virions aggregate to a higher extent (48% of virions were present as double-particle or higher aggregates) than control virions (15%). Bars, 200 nm. (TIFF) [file ppat.1005595.s008.tiff]

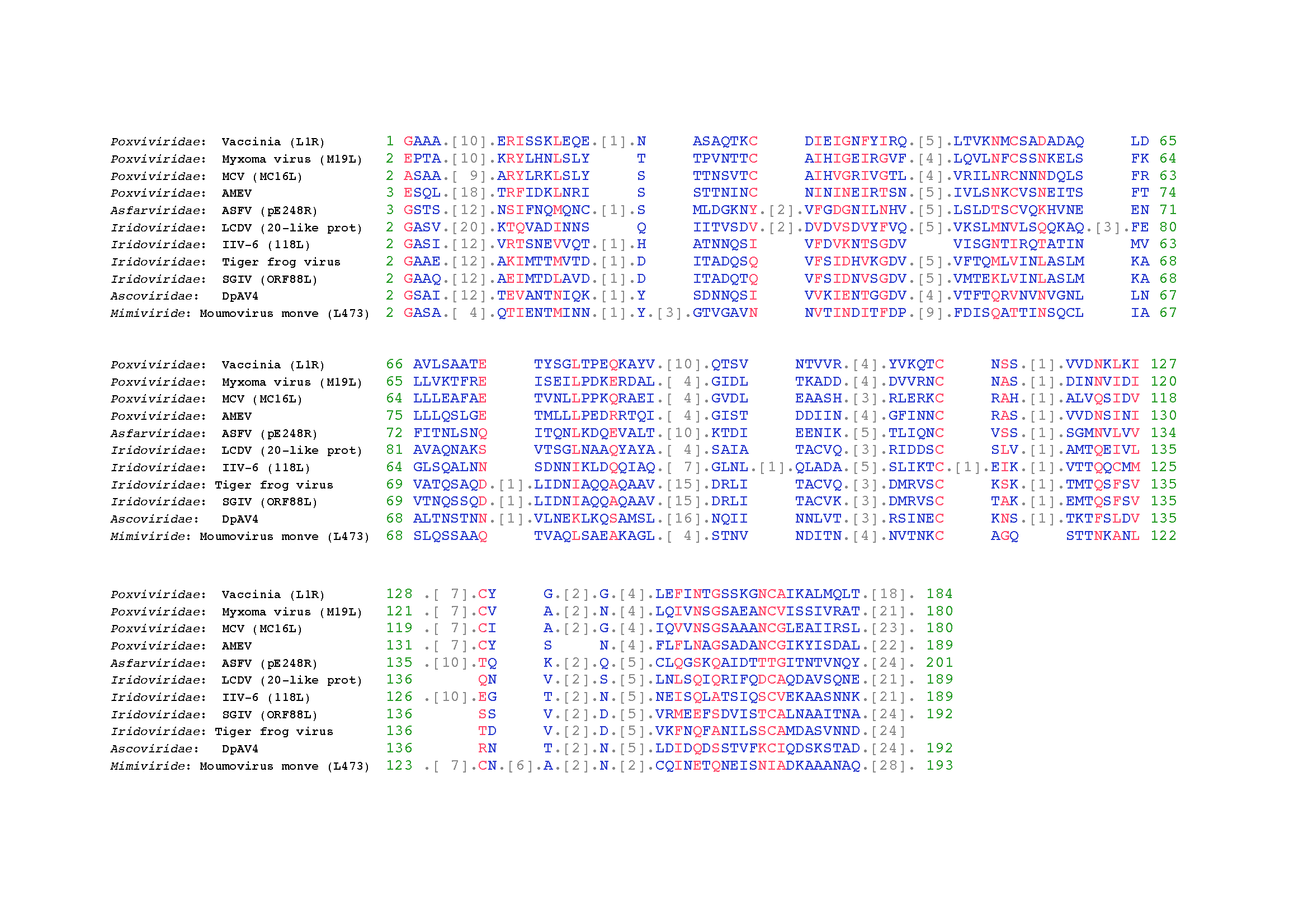

Supplement: S9 Fig — Amino acid sequence alignment of some viral orthologs of VACV protein L1, a member of the entry/fusion complex. The protein cluster (pfam02442: L1R_F9L) includes putative and known transmembrane polypeptides of several NCLDV families like Poxviridae (L1R), Asfarviridae (pE248R), Iridoviridae, Ascoviridae and Mimiviridae. Some selected members of the cluster are shown (complete sequence alignment can be found at http://www.ncbi.nlm.nih.gov/Structure/cdd/cddsrv.cgi?uid=pfam02442&islf=1). Amino acids exhibiting some degree of conservation are shown in upper case, and red color indicates highly conserved residues among rows (default 2.0-bit threshold is shown) while blue indicates less conserved residues. (TIFF) [file ppat.1005595.s009.tiff]
